# ‘Glocalizing’ land-use and forest governance in the tropics: examining research partnerships and international forest policies affecting Brazil, DRC and Indonesia

**DOI:** 10.12688/f1000research.130219.1

**Published:** 2023-02-02

**Authors:** Thiago Kanashiro Uehara, Florie Chazarin, Louise Nakagawa, Ariane Favareto, Tamara Tobias, Arilson Favareto, Rigobert Minani, Bramasto Nugroho, Fitta Setiajiati, Silfi Iriyani, Damayanti Buchori, Chiara Chiavaroli

**Affiliations:** 1 Environment and Society Programme, The Royal Institute of International Affairs, London, Chatham House, 10 St James's Square, SW1Y 4LE, UK; 2 FGV-Ethics, Fundação Getulio Vargas, São Paulo, Av 9 de julho, 2029, 01313-902, Brazil; 3 Research consultant, Environment and Society Programme, The Royal Institute of International Affairs, London, Chatham House, based in, Lima, Peru; 4 Cebrap Sustentabilidade, Centro Brasileiro de Análise e Planejamento, São Paulo, Rua Morgado de Materus 615, 04015-051, Brazil; 5 Centre d’Etudes pour l’Action Sociale, Kinshasa, 9, Avenue Père Boka, Democratic Republic of the Congo; 6 Centre for Transdisciplinary and Sustainability Sciences, IPB University, Bogor, Kampus IPB Baranangsiang, Jawa Barat 16127, Indonesia; 7 Faculty of Forestry and Environment, IPB University, Bogor, Kampus IPB Darmaga, Bogor 16680, Indonesia; 8 Equality, diversity and inclusion consultant, London, UK

**Keywords:** Research partnerships, North–South, forest governance, forest policy, transnational initiatives, international cooperation, Brazil, Indonesia, Congo, DRC

## Abstract

**Background:** International and market forces are key drivers of deforestation and forest degradation, with transnational and market-based solutions in land-use and forest governance often missing economic, distributive, and environmental targets.

**Methods:** This paper tackles both the framing and effectiveness of transnational initiatives affecting forest lands and peoples in the Global South, and the quality of relationships between institutions in the Global North and the Global South. Through more equitable research partnerships, this paper draws lessons from case studies in Indonesia (legality verification system in different forest property regimes), the Democratic Republic of the Congo (lifting of a moratorium on new logging concession), and Brazil (FSC in the Amazon region and the Amazon Fund).

**Results:** International partnerships have privileged market-based instruments and commodity exchange between Global South and Global North countries, and the benefits of such mechanisms are unevenly distributed. Complementary and alternative policy instruments are discussed for each geography.

**Conclusions:** Glocalizing land-use and forest governance implies in advancing equitable research partnerships between institutions in the Global South and Global North, and strengthening a community of practice for critical enquiry and engagement in partnerships for sustainable development. Land-use, climate and forest governance mechanisms must redress power dynamics, and partnership models, and commit to improving well-being and sustainable livelihood outcomes.

## Introduction

International and market forces – particularly industrial-scale commodity markets and large-scale land acquisitions – are key underlying drivers of deforestation and forest degradation, according to the International Panel on Climate Change (IPCC). ^1^ Their latest (2022) assessment comes as international and market-based mechanisms to steer land use in the tropics are continuing apace, and as climate crises compound environmental degradation and systemic poverty. The international community needs to take stock of how it frames and manages land-use, forest and climate policy collaborations: it needs to reflect on lessons learned to date, scale up effective solutions, rethink those that have not worked as intended, and improve the dynamics of transnational collaborations.

A rethink of international collaborations needs to be conducted in an inclusive manner. This includes revisiting Sustainable Development Goal (SDGs) 17 on ‘Global Partnerships for Sustainable Development’, the most central and implementation-focused of all the SDGs and climate targets. ^2^ More inclusive international collaborations entail policy institutes in the ‘Global South’ leading and co-leading on critical analysis of international partnerships, and institutions in the ‘Global North’ acknowledging and learning from diverse perspectives, as well as enabling partnerships for ‘glocal’ land-use and forest governance analysis and solutions.

In this paper, being ‘glocal’ (or ‘glocalizing’) is not about translating, adapting or implementing Global North (or so-called ‘global’) ideas onto the ‘developing’ world. Rather, ‘glocalizing’ relates to redressing imbalances in the interplay between ‘global’ and ‘local’ imperatives in international collaboration, and between institutions in Global North and Global South countries.

This publication brings together the results of a research partnership between four policy institutes: CEBRAP Sustentabilidade ( *Centro Brasileiro de Análise e Planejamento’s* sustainability centre) in Brazil, CEPAS ( *Centre d’Etudes pour l’Action Sociale*) in the Democratic Republic of the Congo, CTSS IPB University (IPB University’s Centre for Transdisciplinary and Sustainability Sciences) in Indonesia, and Chatham House (Royal Institute of International Affairs) in the United Kingdom. ^3^ Through a twenty-month collaborative project, the four institutes sought to establish and discuss equitable research partnerships while examining prominent transnational initiatives that are affecting forest lands and peoples in the tropics.

The results of this work speak to the scientific and international communities – primarily those with an interest or power in reframing international partnerships. ^4^ This paper highlights the importance that should be given to three dimensions of ‘global partnerships for sustainable development’: (i) the quality of the relationships and research partnerships that exist between policy institutes in the Global North and the Global South; (ii) the role of policy institutes headquartered in the Global South in leading or co-leading critical analysis of transnational policy mechanisms that affect the ‘developing world’; and (iii) the need to redress not only power asymmetries but also policy mixes and models to achieve domestic and global priorities for sustainable development, which should go beyond supporting international supply chains, and position well-being and sustainable livelihood outcomes at the core of international partnerships.

The first section ^5^ frames this publication in terms of policy ideas, proposed solutions for and myths about forest governance. It highlights the growth of voluntary and transnational partnerships centred on commodities and international supply chains. The second section ^6^ discusses the process of establishing research partnerships between policy institutes in the Global North and South, in light of principles of equity, diversity and inclusion (EDI). Led by policy researchers in think-tanks based in the countries with the largest tropical forest areas in the world, the third section presents insights and ‘glocal views’ on the Forest Stewardship Council (FSC) and the Amazon Fund in Brazil, ^7^ on World Bank-led reforms that are affecting logging concessions in the Democratic Republic of the Congo (DRC), ^8^ and on the relevance of a timber verification system for different forest regimes in Indonesia. ^9^ Note that domestic audiences in Brazil, the DRC and Indonesia were reached through policy briefs published in their preferred languages. ^10^ The conclusions revisit the nature of prevalent land-use and forest solutions, with recommendations for the international community of practice and research on climate policy, land-use and forest governance.

## Forest governance solutions

Despite the absence of an effective global agreement on forests, international institutions, transnational banks, philanthropies and donor governments have supported various North–South initiatives aimed at reducing or halting deforestation and forest degradation tout court in the tropics. Such initiatives include those tackling illegal logging and its related trade, sustainable forest management and governance reforms in low- and middle-income countries. ^11^ Multilateral coordination on climate and forests seems to be growing. Some of the latest United Nations Conferences of the Parties (COPs) have launched several voluntary, multiparty pledges, such as the Glasgow Leaders’ Declaration on Forests and Land Use, and the Global Forest Finance Pledge at COP26 in Glasgow, and the Forest and Climate Leaders’ Partnership at COP27 in Sharm El-Sheik.

Voluntary initiatives (self-determined or self-imposed), incentive-based instruments and market-based solutions have been growing as forest governance mechanisms. ^12^ Such initiatives have been on the rise since the 1990s, ^13^ and include the FSC, REDD+, ^14^ Voluntary Partnership Agreements (VPA) between the EU and timber-producing countries, and economic recovery loans by the World Bank conditional on reforms in resource management. The role of the private sector in shaping forest governance solutions further expanded in the 2000s, with the establishment of initiatives such as the Tropical Forest Alliance, commodity roundtables, the Soy Moratorium, and the Consumer Goods Forum deforestation pledge – all of which mirror the evolution of corporate social responsibility and sustainability in supply chains. Corporations (focal firms like Unilever, Nestlé, Cargill, Klabin or Ikea) have for some decades addressed sustainable supply chain management, ^15^ with a growing role for environmental non-governmental organizations (NGOs) in supporting both private and public initiatives for sustainable supply chains. ^16^

Governments and focal countries have been following suit by complementing or supporting market-based mechanisms and solutions in sustainable supply chains – for instance, through demand or import regulations and due diligence mechanisms for deforestation-free supply chains. The US Lacey Act, as amended in 2008, and the EU Forest Law Enforcement, Governance and Trade (FLEGT) action plan established in 2003 are pioneers in engaging governments in the Global North in supporting ‘sustainable supply chains’. ^17^ All these initiatives, which include the more recent pledges at COP26 and COP27, fall under the umbrella of official development assistance (ODA) and SDG 17. ^18^

### The need for critical analysis

Some of the prevalent ‘solutions’ for improving forest governance are frequently not backed up by available scientific evidence, according to a 2020 review by Delabre *et al*., ^19^ For instance, one myth is that ‘markets are the solution to deforestation and forest degradation’, with forest politics emphasizing voluntary agreements and market mechanisms ^20^ as prevalent and growing forest governance mechanisms. This myth usually comes with the assumption that market-based mechanisms are more efficient and cost-effective than regulations. ^21^ Yet, the assessment of ‘efficiency’ changes when social indicators such as well-being are prioritized over economic costs. ^22^

In assessing sustainability in supply chains, the challenge is not only to identify how a commodity is produced, traded and consumed, but mainly to find the convergence of expectations between buyers and suppliers when it comes to defining ‘sustainability’, and distributing benefits across value chains. ^23^ Transparency and open-data enterprises have helped such assessments, particularly when supported by technological innovation, big data ^24^ or participatory and independent monitoring systems. ^25^ These mechanisms facilitate sustainability assessments of industrial modes of production, and export-oriented and import-dependent economies, but rarely address well-being or inequality indicators, ^26^ or risks of leakage ^27^ (collateral effect across governance boundaries). ^28^ For instance, export-oriented production systems (such as financial and industrial forest and agri-food practices) have been better positioned to benefit from sustainability verification systems than domestic or ‘civic’ production systems, when these are based on shorter circuits of commercialization, cooperation or reciprocity logics. ^29^

Solutions also prove inadequate when targets, indicators and metrics of so-called ‘good’ forest governance are oversimplified and overlook dynamics of corruption, oppression and marginalization. ^30^ For instance, another known myth influencing decisions at funding, development and implementing levels, is the assumption that local communities are already included in decision-making. ^31^ Efforts to include smallholders into international and market initiatives have had only limited results and success. ^32^ Heterogeneity and intersectionality are often oversimplified in ‘participatory’ forums, especially beyond the design phase of an initiative. ^33^

In sum, the results of forest solutions have varied from limited impacts to selective reforms in governance, with the distribution of costs and benefits of prevalent solutions rarely aligning with principles of sustainability and equity. ^34^ Despite the sum of efforts invested in public regulations, private-led initiatives and hybrid forms of forest governance, the world’s forest area continues to shrink. This area includes the countries with the largest expanses of tropical forests – Brazil, the DRC and Indonesia – where rural and forest peoples struggle for autonomy amid varying levels of poverty, inequality and market integration. Selected indicators of the state of forests and the biggest tropical forest countries are summarized in Table 1.

**Table 1.** Selected global and country-specific indicators on forests, governance, poverty, inequality, and trade.

	Global	Brazil	DRC	Indonesia
Forest policy and governance ^35^	Inadequate/ insufficient	Fair	Weak	Fair
Forest cover ^36^ ^,^ ^37^	4.06bn hectares 31% of land area	497.8m hectares 60% of land area	127.3m hectares 56% of land area	92.7m hectares 49% of land area
Principal direct driver of deforestation ^38^	Forestry, commodity-driven deforestation	Commodity-driven deforestation	Shifting agriculture	Commodity-driven deforestation
Multidimensional poverty ^39^	1.2bn people across 111 ‘developing countries’ 19.1%	8.2m people 3.8%	59.9m people 64.5%	9.8m people 3.6%
Inequality: Top 10% net personal wealth share ^40^	The wealthiest 10% owns 77% of total wealth	The wealthiest 10% owns 80%	The wealthiest 10% owns 62%	The wealthiest 10% owns 61%
Forestry commodities trade, 2020 ^41^	$154bn 3.2% of all commodities trade	$10.4bn 6.8% of global forestry products trade	$0.1bn <0.1% of global forestry products trade	$6.3bn 4.1% of global forestry products trade

‘Governance responses to addressing the direct and underlying drivers of deforestation have been inadequate to reduce pressures’, according to the 2022 IPCC assessment on forest governance. ^42^ It notes that transformations are needed in several areas, such as the strengthening of environmental laws and policies, better combination of public, private and hybrid initiatives for environmental responsibility, acknowledging land tenure and rights, and enhancing inclusive stakeholder participation to ensure equitable outcomes. ^43^ Also, as remarked by Lambin *et al*., privately driven mechanisms have the potential to address regulatory gaps in land-use governance in the tropics under ‘appropriate policy mixes’, where ‘various policy instruments perform complementary functions without undermining each other’. ^44^ Notably, ‘public regulations maintain an essential role of protecting basic environmental conditions, as well as providing the enabling conditions for private and hybrid initiatives, and pushing standards upward’. ^45^ In order transform land-use and forest governance responses, it is essential to redress relationships and partnerships between actors across the Global North and South.

Prior to presenting the analysis led by policy researchers with CEBRAP, CEPAS and CTSS in partnership with Chatham House (Section 3), the next section looks at the challenges and lessons learned in establishing research partnerships between these policy institutes. By doing so, we hope to enable greater political accountability and more inclusive governance, which can be seen as part of a ‘transformative lever towards improving environmental governance and resilience of tropical forests’. ^46^

## North–South and South–South research partnerships

Efforts to redress, diversify and enrich local and global perspectives in international cooperation and in research partnerships should pass through the implementation of equitable North–South partnerships. In this section, we argue that the construction of ‘equitable’ research partnerships is a fundamental dimension of the set of North–South relationships to be established in order to improve environmental governance and resilience of tropical forests. The section includes a critical analysis of conventional modes of North–South relationships, as well as the challenges and lessons learned in building a North–South and South–South research partnership between CEBRAP, CEPAS, CTSS and Chatham House. This reflection has the potential to inform good practices in transnational climate and forest collaborations given the limited scholarship that discusses equality, diversity and inclusion in North–South research partnerships in resource governance. An emerging debate on ‘decolonizing’ research invites scholars to employ equitable publication policies and participatory methodologies, and to consider the power relationships that emerge with other researchers and research participants. Yet, neocolonial practices still significantly shape North–South research partnerships, which were avoided in this project.

The geopolitical power asymmetry between the Global North and the Global South is often mirrored in transnational research partnerships. ^47^ Traditionally, research partners in Global North countries have disproportionate power across several areas of a research partnership, such as: decision-making arrangements; research design; authorship arrangements; and funding management. The asymmetry of power is commonly manifested when Global North partners design and control the entire research process, while their counterparts in the Global South collect data, with limited to no say in research design, methodology and research outputs. The Global South is often treated as ‘a laboratory for the North providing interesting scientific data’, in the words of Maselli *et al*., ^48^ with institutions in the Global South not enabled or allowed space to participate effectively in knowledge production. Notably, these research practices lead to efficient and systematic manners to extract and appropriate knowledge from research participants and local partners, with this knowledge rarely reaching or benefiting local participants and national stakeholders.

Before beginning a collaboration with CTSS, CEPAS and CEBRAP on (g)local forest governance in 2020, Chatham House had worked on global forest governance for two decades. The idea to advance equitable partnerships emerged after discussions at the long-standing series of Global Forums on Forest Governance organized by Chatham House, ^49^ and from the identification of the need to enable and embrace greater diversity as a transformative lever that improves policy analysis and solutions. The opportunity identified was to craft and initiate an innovative work programme to work with and learn from researchers and analysts headquartered in policy institutes in some of the countries with the largest tropical forests in the world. This idea was discussed between Chatham House researchers and funders, and implemented in the 20 months from March 2020 to the end of 2022. Although some of the institutions were known to Chatham House through other projects, like CEBRAP and IPB University through the Trade Hub/UKRI consortium, the relationships with CEPAS and CTSS were crafted from zero.

### Setting up relationships

A clear set of ground rules on the relationships among partners was established in this project. A first, important step to construct new and more equitable practices was to recognize and openly discuss the asymmetries of power derived from Chatham House’s privileged relationship with funders, which resulted in Chatham House writing and negotiating the proposal, defining its overall scope and allocating budget. Second, an anonymized Equity, Diversity and Inclusion (EDI) survey was conducted in the first months of the partnership, with all researchers involved in the project, to identify how they understood responsibilities and roles in the decision-making process. Arnstein’s ladder of participation ^50^ – a framework that describes and identifies different types of participatory spaces ranging from no participation to effective participation – was used to map current modes of relationship and clarify internal decision-making arrangements. Rather than creating a space of ‘formal’ participation, it was important for all partners to clarify which elements of the project could be negotiated and which could not, since they were already established in the agreement between Chatham House and the funder. Discussing this openly avoided forms of ineffective participation such as ‘tokenism’ and ‘manipulation’. ^51^ Moreover, each participant identified how much decision-making power they were aiming to have in the project and what effective participation meant to them.

One important outcome of the survey was that partners openly negotiated and agreed on the establishment of independent spaces of decision-making with the aim to preserve the autonomy of each policy institute within the limits of the project proposal. In particular, for researchers based in the Global South, it was important to have the autonomy to select their case studies, research methodologies and key literature including scholars writing in languages other than English, such as Portuguese, French and Indonesian. This practice differs from common processes which limit agency of researchers in the Global South. The agreed balance between dependence and independence varied by policy institute, according to the capacities, traditions and preferences stated by the institutes in the Global South, as well as the limits posed by the terms of references agreed between Chatham House and funders. There was no single solution in partnership management, and adaptability was key for Chatham House to accommodate differences within the limits of the project objectives, budget and timeline. Based on the results of the survey, Chatham House staff adjusted project management practices to support more autonomous decision-making in the project. In particular, it was important for Chatham House (CH) staff to recognize that the partners felt the exercise of subtle coercion (later discovered as unintended) when suggestions and recommendations were made by CH staff. This arose both in light of the privileged relationship of Chatham House with the partners and on the basis of implicit assumptions related to its authority as a renowned institution in the UK and in the Global North in general. CH staff were not aware of the coercion felt by partners, particularly when using the verb ‘to encourage’ in meetings. But this was discussed with the EDI consultant, leading to CH staff revisiting their ‘positionality’, ^52^ rethinking practices when providing comments and suggestions to partners, and understanding the impact they had on partners’ decisions. Notably, partners had a permanent space to raise grievances or complain about decision-making processes in the project.

At the end of the project, another anonymous survey revealed positive feedback by the partners in the Global South with regards to the management of the project’s decision-making processes, and an appreciation that their inputs and suggestions were collected by an independent consultant and then shared with, and enacted by CH staff. In particular, all participants agreed that decision-making arrangements with Chatham House oscillated between equal decision-making and the co-definition of the research objectives and methodologies, with substantial responsibilities delegated to the partners.

South–South collaborations are often overlooked when compared to North–South research partnerships, ^53^ but this project sought to address this gap. In this research, similarities and differences in the political economies of Brazil, the DRC and Indonesia were acknowledged, *vis-à-vis* domestic development challenges and the international pressures. The overall assessment was that transnational mechanisms have largely benefited international operators (i.e., donors, banks, multinational companies) rather than local or national stakeholders, and that there was a case for this dynamic to be redressed.

Also, the assumption was that policy institutes in the South would benefit from strengthening ties. For this reason, channels for South–South collaboration with and without mediation by Chatham House were established. Partners in the South engaged in bilateral meetings, presented their results to each other, and exchanged comments on each other’s work. These were important steps to strengthening capacities for collaborations, and to build a collective critique of transnational policies.

### EDI in methods and outputs

The inclusion of local knowledge is fundamental to socially just environmental policymaking. ^54^ The inclusion of women, Indigenous Peoples (IP) and local communities in knowledge co-production is considered key to tackling the environmental risks related to climate change. ^55^ Mindful of this, research partners engaged in a critical reflection on their positionality and on the ways in which their institutional identity could frame project outputs by privileging certain voices over others. It was important to recognize how the political and religious identity of the institutions involved in the project implied the exclusion of certain local voices, and how to deal with this within the research process, and to acknowledge both personal and institutional positionality.

In terms of frameworks and guidance, the researchers discussed diversity and inclusion in research methodology, inclusive terminologies and classifications of agents in forest governance, and the need to use gender-sensitive focus groups or to include questions on gender, ethnicity and age as determinants of access to natural resources. This also implied critically assessing the extent to which the project participants who consulted in the research represented the diversity of local stakeholders. Finally, in the process of analysing the research results, particular attention was given to the distributive aspects of forest governance initiatives.

An effort was made in relation to the adaptation of the research outputs to formats that allowed more incidence in domestic contexts and among diverse stakeholders. This was attempted by producing policy briefs in different languages (Bahasa Indonesia, French and Portuguese) and audio-visual materials to share the project results with important stakeholders who do not belong to elites.

To conclude, addressing power asymmetries in research partnerships is necessary for implementing socially just practices of knowledge production. Openly discussing project management practices helped advance equitable collaborations, opening up an important space of reflexivity for institutions and researchers working in transnational agreements. Finally, an EDI toolkit on establishing equitable research partnerships can be found as a publication in the Chatham House Forest Governance and Legality website. ^56^

## (G)local views on transnational policy instruments

This section examines transnational forest governance mechanisms followed by insights and recommendations from policy researchers in the Global South, with CEBRAP, CEPAS and CTSS. Each policy institute examined transnational mechanisms understood as influential and paradigmatic ^57^ (cases that highlight general characteristics of transnational mechanisms affecting land-use and forest governance).

The FSC and the Amazon Fund are investigated in Section 3.1, led by Louise Nakagawa and CEBRAP colleagues in Brazil. The implications of a World Bank economic recovery loan to the DRC, conditioned on new regulations on forests and logging concessions are discussed in Section 3.2, led by Rigobert Minani with CEPAS. Indonesia’s timber legality and sustainability assurance system, which was co-sponsored by the EU, is discussed in Section 3.3, led by Bramasto Nugroho and CTSS IPB University colleagues.

Box 1. **Initiatives in brief.**

The **FSC** is an international non-profit, multi-stakeholder organization created in 1993 in response to the failure of the 1992 UN Earth Summit in Rio de Janeiro to reach a global agreement to stop deforestation. ^58^ As an alternative to boycotting forest products, the FSC proposed a voluntary market-based instrument (eco-certification) to improve forest management. The FSC gained a reputation as an ecolabel and certification programme. ^59^ Of the total FSC-certified area, 4% is owned by smallholders. ^60^

Primarily funded by Norway and Germany, the **Amazon Fund** is one of the first UN REDD+ mechanisms. ^61^ The notion of REDD – Reducing Emissions from Deforestation and forest Degradation – first emerged in climate negotiations. The role of conservation, sustainable forest management and forest carbon stocks in developing countries were added to become REDD+, in 2008. ^62^ REDD+ is ‘the largest payment for ecosystem services initiatives worldwide’, ^63^ and it provides incentives for countries to seek and obtain results-based payments based on carbon dioxide (CO _2_) metrics.

Forest concession is a contractual arrangement for the temporary allocation of public forest resources to another party, such as companies, communities and NGOs. ^64^ Forest concessions have been widespread across tropical regions, ^65^ including the DRC, but in 2002 a **moratorium** (which is a legal temporary prohibition or suspension of an activity) was imposed on new concessions. ^66^ The moratorium, which was encouraged by the World Bank, was expected to help the DRC to control land use, collect revenues and repay debts. ^67^

Due diligence and **verification mechanisms** are gaining popularity as part of the commercialization of forest and agricultural commodities. Indonesia's national timber sustainability assurance system, SVLK (Sistem Verificasi Legalitas Kayu), is one of the first of its kind. Launched in 2009, SVLK is a key component of the FLEGT Voluntary Partnership Agreement established between Indonesia and the EU. It aims to ensure that timber is harvested, transported and processed legally. ^68^

Notably, the FSC, Amazon Fund, the moratorium on concessions, and verification mechanisms have international or transitional origins; they tackle commodity markets (i.e., timber and carbon); and were adapted and implemented in multiple countries. FSC-member organizations are present in 89 countries; ^69^ 16 countries submitted REDD+ action plans to the United Nations Framework Convention on Climate Change (UNFCCC); ^70^ Central African countries have followed the World Bank’s conditionalities on reforming forest codes; and 15 countries are implementing or negotiating FLEGT Voluntary Partnership Agreements with the EU, which requires the establishment of a timber legality verification system. The analysis that follows should by no means be generalized but is particular to each specific context, and it should be read in the context of the glocalization of forest governance analysis and solutions.

## Revisiting community and governance impacts of the FSC and Amazon Fund in Brazil

The Amazon is a natural heritage region for humanity. It plays a vital role in regulating the global climate system, ^71^ besides being responsible for the rainfall regime covering a sizeable portion of Brazil. ^72^ Its forests contain the highest biodiversity on the planet, ^73^ and are home to numerous peoples and ethnic groups. ^74^ However, over the past decades, the biome has suffered severe impacts due to uncontrolled and illegal use of its natural resources, resulting in the advance of deforestation. Between 2005 and 2012, deforestation rates decreased in the Amazon thanks to the combination of strong public policies and voluntary market mechanisms. But the situation has reversed since 2012, and in 2021, under Jair Bolsonaro’s presidency, the biome had its largest forest loss since 2006.

In addition to the negative impacts on the environment and the climate, the Amazon region has the highest concentration of poverty in Brazil. ^75^ It is the only region in the country where inequality indicators, such as electricity and health access, deteriorated during the first decade of this century. ^76^ In light of the increasing deforestation rates, the escalation of conflicts over land, the increased vulnerability of rural and forest peoples, and the impoverishment of the most vulnerable populations, what paths have been built to strengthen forest governance for the Brazilian Amazon?

This section revisits two of the most influential mechanisms affecting land use and livelihoods in and around forests: the Forest Stewardship Council (FSC) and the Amazon Fund as a REDD+ mechanism. The first is primarily focused on promoting better forest management practices, and the latter on reducing emissions and carbon markets or payment for environmental services. ^77^ Notably, multiple sources show that the causes of long-term deforestation or the promotion of the well-being of forest peoples have not been sufficiently tackled through such instruments. ^78^ Examining such mechanisms is even more important under current circumstances – namely, rising deforestation rates and inequality in the Brazilian Amazon ^79^ – as well as for the lessons learned that can inform the next mandate of Lula da Silva’s presidency. Thus, considering the governance structures, achievements of and controversies relating to the FSC and Amazon Fund in the region, what can we learn and recommend for the future of international cooperation on forest governance?

To explore this question, we conducted a literature review, workshops and key informant interviews with representatives of the government, NGOs, funding agencies, groups of IP holding FSC certification, and beneficiaries of the Amazon Fund. ^80^ On the basis of this, we reached a few conclusions and observations about both mechanisms.

### Varying well-being benefits and (selective) inclusion

The FSC contributes to material well-being and productivity of certified units managed by IP and forest peoples, helping to boost incomes and structure non-timber forest product (NTFP) chains. However, the FSC has several limitations, particularly for smallholders, Indigenous Peoples and local communities. The FSC is a privately led initiative, and its effectiveness in forest enterprises led by forest communities and other marginalized groups depend substantively on external support, which is often absent. In our research, we verified that smallholders are dependent on supporting entities (either government or more recently private sector) to obtain and maintain FSC certification. Besides lack of support for minoritized groups, interviewees stated that smallholders face constant threats and incursions by land grabbers – a situation that has worsened during Bolsonaro’s presidency, due to weaknesses in monitoring, command and control mechanisms, and government inertia (or in some cases, encouragement of illicit deforestation and illegal logging). It is indeed very challenging for IP, local communities and smallholders to obtain and maintain FSC certification, since its standards and procedures are not easy to navigate or comply with. The insufficient levels of credit and financing for smallholders do not help them to finance FSC certificates. The low levels of support to smallholders in Brazil stands in stark contrast to the budget allocated by the federal government to agribusiness, mining and major infrastructure works.

The Amazon Fund, which received donations from the governments of Norway and Germany, and to a lesser extent from Petrobras (Brazilian oil and gas company), has enabled grassroots organizations, NGOs and community associations to build and strengthen capacities to network and partner in international cooperation. The Amazon Fund contributed to strengthening environmental agencies at the subnational (state) and national (federal) levels. Even when Bolsonaro froze the Fund and dismantled its participatory governance aspect, the legacy of the Amazon Fund was noticeable through the continued efforts made by institutions that it had strengthened in the past. One of the most important impact areas of the Fund ^81^ is the combination of positive outcomes for both forest conservation and the promotion of community well-being and safety of forest peoples. Amazon Fund initiatives are often associated with the inclusion of women and traditional communities – such as IP and *Quilombola* communities – in decision-making processes, which have been more respectful of their livelihoods, cultures, knowledge and values.

When both cases are analysed, using the Institutional approach, ^82^ we observe that the effectiveness of forest governance is compromised in two ways. These are when stakeholders’ participation is limited, such as by excluding the government, as in the case of the FSC; and when peasant family farmers, IP or local communities are further marginalized. In circumstances where there are low levels of participation, autonomy and empowerment among local stakeholders, a discontinuity of initiatives, dependency and marginalization linked to poor well-being outcomes can be expected, not only in terms of productivity, access to markets and public policies, and income, but also in health, education, institutional capacity, safety, and food and land security.

### Lessons learned and recommendations

Both the FSC certification and the Amazon Fund have been important forest governance mechanisms for the Amazon. However, they cannot be considered as unique or sufficient solutions. Both have generated positive impacts on people’s lives, albeit in different domains, but there is still much room to discuss new strategies and actions that governments, funders and investors can enact at national and international levels.

Despite efforts to implement forest governance mechanisms that address sustainability and inclusion at the same time, specific topics still require more attention. Capacity-building is one of them. Actors and institutions depend on knowledge, networks, visibility and capacity for engagement in order to compete and get access to technical and financial support.

Another topic relates to participation. It is crucial to place local and minoritized groups at the centre of decision-making. Listening to local communities, NGOs and subnational governments have meant that funds are directed and spent in a way that respects and values the cultures and livelihoods of diverse populations – who feel more empowered and able to improve their living conditions. At the same time, this approach, which was embedded in the Amazon Fund, has contributed to the enforcement of important instruments of command and control. With Lula returning as president, and the prospects that the Amazon Fund could be co-managed by the central and subnational governments in collaboration with civil society, Norway has committed to resume donations to the Fund.

Coordination between state and non-state actors is therefore key to designing, implementing and improving policies that integrate the social and environmental agendas, promoting sustainability and inclusiveness in the biome. In this sense, international cooperation has a great role to play, helping to build an open, trusting and pragmatic space for negotiation between governments, the private sector, funders, banks, investors, grassroots and civil society organizations.

To achieve this, we recommend that international development funders such as the EU and UK governments create the necessary conditions to enable and broaden out forums for equitable, diverse and inclusive participation of Global South agents and institutions in their forest governance programmes. These should include language interpretation, the right to veto and consideration of local priorities such as food and land security, or the development of solidarity and well-being economies in and around tropical forests. We recommend that government funders also negotiate the implementation of glocal mechanisms that consider and respect national sovereignty.

To banks and investors, our recommendations are: i) to support local/regional initiatives that respect, value and promote sustainable livelihoods and local knowledge as a premise, as well as the inclusion of marginalized populations in just and sustainable supply chains, to the extent that such populations want to engage in such markets; and ii) to halt investments and financing of activities that impact negatively on forest conservation and put at risk the livelihoods and human rights of those in and around the Amazon.

Moreover, we recommend international cooperation to update theories of change and monitoring, evaluation and learning (MEL) processes. The social dimensions of forest governance should be thoroughly integrated in theories of change and MEL plans, with the establishment of appropriate indicators and metrics capable of measuring inclusion and well-being, as suggested by Shaafsma and others. ^83^ Reconsidering sustainable livelihoods frameworks linked to political economy analysis could also help rethink and reshape international cooperation. ^84^ The expected impacts, outcomes and targets of forest governance mechanisms must go beyond the legal, biophysical or environmental dimensions.

### Final remarks

To achieve successful glocal forest governance, ‘global’ initiatives must explicitly incorporate local perspectives through inclusive and participatory design and implementation. International organizations would benefit from ‘glocalizing’ their approach and operations, recognizing the complexities of diverse biomes, and encouraging, sponsoring and listening to independent platforms that convene different economic sectors and groups in society. Our recommendations would contribute to better coordination and deliberation processes. Notably, this will only lead to fair and sustainable outcomes if done through an inclusive coalition that works on territorial or place-based development agendas to address root causes of deforestation.

Finally, we highlight three considerations for policymakers in international cooperation. First, integrate bottom-up and top-down mechanisms into policy strategies. Dialogues must consider the pluralities of situations and local realities. Second, foster and strengthen forest governance arrangements built on inclusive and transformative coalitions. And last, but not least, develop strategies and actions that transcend environmental sustainability or legality, adding the much-needed elements of inclusiveness and socio-economic targets and indicators to the international cooperation agenda.

## Regulating logging concessions and the moratorium in the DRC

In July 2021, the government of the Democratic Republic of the Congo lifted a ‘temporary prohibition’ – or a moratorium – on the allocation of new industrial logging concessions, which had been in place for 19 years, since 2002. Different stakeholders – whose views we explore in this section – saw this as both an opportunity and a risk.

The moratorium on new logging concessions was intended to put an end to ‘widespread looting, exploitation, and destruction of the country’s vast forests’. ^85^ It proved to be largely ineffective, given the current levels of deforestation and illegal logging. ^86^ One could ask, therefore, whether the moratorium should be continued or lifted?

To put this policy change in context: the DRC negotiated a peace agreement to end the Second Congo War in 2002, along with financial support from the World Bank, for a post-conflict economic recovery plan. This financial support was conditioned on reforms over the regulation of mining and forests, ^87^ which the DRC government quickly responded to, having promulgated new codes in 2002. ^88^ ^,^ ^89^ This change mirrored what other Congo basin countries had gone through in previous years, such as Cameroon in 1994, following conditionalities by the World Bank. Limited consideration was given to the impacts of such changes on IP, local communities or the environment. ^90^ Capacities of local governments were perceived to be at risk. ^91^ Nevertheless, legal reforms were made, with the 2002 forest code expected to promote sustainable forest management, contribute to increased government revenues, and help the country to ‘develop’ and build capacity to repay debts.

The conditions for lifting the moratorium were only established in 2015, after a presidential decree. The conditions were three: i) conversion of old logging titles into forest concession contracts; ii) deployment of transparency mechanisms on forest concessions; and iii) plan for a phased lifting of the moratorium.

In 2021, although these conditions had not been fulfilled, the government announced the lifting of the moratorium. In 2022 the government also announced the auction of new oil exploration blocks, which would affect forests and peatlands. As articulated by international NGOs, these government decisions were at odds with the DRC’s stated aim to become a ‘climate crisis solution country’. ^92^ Among these perceived contradictions, we examined the views of different stakeholders about the moratorium on industrial logging concessions in the DRC, in order to find ways forward in terms of national and international policy work. We gathered the perspectives of various stakeholders in workshops and interviews in Kinshasa and in the province of Tshopo, including representatives from IP, Congolese civil society, international NGOs and the DRC government. ^93^ Their views should all contribute to advancing the glocalization of forest governance solutions and analysis.

### Views on the moratorium on industrial concessions

For the DRC government, one of the main reasons to lift the moratorium on industrial forest concessions is that maintaining a 20-year-old moratorium is ‘irresponsible’. ^94^ DRC officials emphasize the potential for land resources to contribute to revenue collection for the government, to the development of infrastructure, and to the benefit of people. According to the DRC’s General Director of Forests, the DRC ‘is home to 60% of forests in the Congo basin, with strong timber potential – of above 10 million cubic metres per year, and a huge farming potential, of around 80Mha of arable land’. ^95^ These resources should be mobilized, the General Director argues, which would be crucial to fulfil the needs of a growing population and infrastructure development. Biodiversity conservation or mitigation of climate change are not evident in this viewpoint.

International NGOs and Congolese civil society differ from the government. Some international NGOs denounced a contradiction between the DRC’s climate and forest commitments and its plan to lift the moratorium on new logging concessions. ^96^ International NGOs called attention to the fact that the main beneficiaries of new industrial concessions in the DRC would be foreign companies, and that this would not necessarily create a trickle-down effect. Greenpeace Africa and Rainforest Foundation UK, for instance, submitted a petition to ‘the international community’ calling for action to stop the lifting of the moratorium. ^97^

Congolese civil society groups, such as the *Groupe de Travail Climat REDD+ Rénové* (GTCRR) and the National Coalition Against Illegal Logging (CNCEIB), argue that the DRC still needs to design and define a phased programme to progressively (and responsibly) lift the moratorium – as per the third conditionality created in the 2015 Presidential Decree. This option would respect the rule of law. Congolese civil society has also called for this process to be done through participatory and multi-sectoral means.

Among representatives of IP, some expressed the hope that the allocation of new forest concessions could be helpful if logging companies contributed to the provision of services to local communities, such as education, health, water and transport. Yet grievances against industrial loggers remain widespread, and IP’ representatives underline the need for any new enterprise to respect social obligations and human rights, such as observing Free, Prior and Informed Consent (FPIC). Our research however, did not explore alternatives to industrial concessions, such as community forest concessions, which have legal underpinnings and support from IP and local communities’ advocates.

We acknowledge that views change, and that differences exist not only between but within groups of actors. This caveat acknowledged, we presented the policy research findings to contribute to the debate in this sector, which is still lacking in data availability, transparency and platforms for dialogue.

### Rule of law and development challenges

Forest law enforcement is weak in the DRC. ^98^ Despite the 2002–21 moratorium on the expansion of industrial logging, the DRC’s forest administration granted contracts through bilateral negotiations between entrepreneurs and government officials. ^99^ Not only were these were unlawful, they also lacked transparency, fairness or competition that a public action should provide.

The General-Inspectorate of Finance (IGF) of the DRC concluded that logging concessions in the two decades between 2000 and 2020 were linked to corruption and benefited personal interests – with consecutive ministers violating the moratorium and failing to facilitate revenue collection. ^100^ Law enforcement has been selective and arbitrary, with some Ministers enforcing certain laws, while others striking deals at their discretion, according to the IGF. ^101^ Unlawful concessions deprived the government of security deposit payments, which would have offered some protection against defaulting operators. ^102^ As a result, logs produced in the DRC – albeit in quantities far below other countries in the Congo basin – are more likely to involve illegality now than at the start of the century. ^103^

The DRC’s capacity to enact reforms or enforce regulations remains limited. The civil service, for instance, faces numerous challenges, such as poor infrastructure, limited headcount or training on environmental policy, forest resources or land rights. Poor infrastructure limits local capacity for timber processing. There is no political and policy coordination to reconcile risks and opportunities between urbanization, rural development, food, energy and climate security. Regulating control and access to land and natural resources is difficult, in general, and this challenge is even greater in low-income countries with weak governance systems, like the DRC.

### Final remarks and recommendations

The arguments of different stakeholders in the exploitation of natural resources in the DRC tackle ecological, climate and local development challenges, and these have not yet been addressed in a systemic manner. International stakeholders have prioritized support to environmental protection in the tropics, including in the Central African region. This is at odds with some of the local priorities and most pressing development challenges, such as the need for rural livelihoods to be strengthened, and for infrastructure and institutional capacities to be built. In our view, international partnerships and finance have encouraged or prescribed standardized and unsuitable frameworks for the DRC, having failed to truly collaborate in understanding and addressing basic needs, local priorities and the concerns of the majority in the Congo basin.

Future international partnerships should aim to, first, learn about the local needs of affected communities, engage with local policy institutes, and then rethink reforms, regulations and action plans. Second, they should encourage coordination between different ministries, for policy coherence. This should be primarily focused on sustainable livelihoods or the well-being of people while also targeting commodity production, trade and environmental targets. Third, international partnerships should support participatory, inclusive, non-partisan design of roadmaps and cross-sectoral plans for sustainable development, and encourage co-management practices for policy delivery with domestic institutions, IP and local communities. Inclusive and deliberative in-country processes, with high levels of quality participation of key stakeholders, including marginalized constituents, should be enabled for the benefit of the forests in the region.

The moratorium on new logging concessions may have stemmed the authorization of mass industrial exploitation of forest lands but it has not stopped illegal practices. Nor has it paved the way for the kinds of governance reforms that the World Bank expected for the economic recovery of the DRC.

How the lifting of the moratorium plays out will depend on the rules that govern new concessions – which need to be revised – and the capacity for law enforcement, monitoring and learning at the country level. Several questions will also need answering, for instance, who would be entitled to manage new concessions? There would be a substantial difference in distributive outcomes if regulations encouraged arrangements like community forest concessions, and if the international community supported business developments led by local producers’ associations, small and medium enterprises. What technical standards for sustainable resource management should be embedded in future land and forest allocations? What would be the role of state agents, grassroots and civil society organizations in monitoring and evaluating such policies and practices? These issues should be addressed soon, in open and transparent forums, so as to contribute ideas and solutions to advance the DRC as a true ‘climate crisis solution country’.

## Verifying legality in different forest regimes in Indonesia

Multilateral frameworks to improve forest law enforcement, governance and associated trade have been supported since the 2000s by the World Bank, the United Nations Forum on Forests, the Group of Eight (G8 – currently the G7), the EU, and the Association of Southeast Asian Nations (ASEAN). ^104^ The hope was that reducing illegal logging and the related trade would halt deforestation and would foster sustainable forest management while improving rural livelihoods and supporting sustainable development. ^105^

In 2016, Indonesia became the first country in the world to operationalize a Forest Law Enforcement, Governance and Trade (FLEGT) licensing scheme, ^106^ which is supported by a verification system called SVLK ( *Sistem Verifikasi Legalitas Kayu*). The SVLK has had several changes since its inception. The major change accompanied the 2021 Omnibus Law on job creation. This change broadened the scope of the system, to encompass verifications on sustainability (beyond legality), and to cover all timber and non-timber forest products, and all forest property regimes. ^107^

There are several studies about the SVLK as a whole, or assessing it from the importers’ perspective. ^108^ This section brings more of a glocal perspective to the matter, focusing on the effectiveness of the SVLK for Indonesia in different property regimes: state, customary and private forests.

Most forested areas in Indonesia (93%) are the property of the state. State forests have been primarily exploited by large companies through concessions for industrial logging and timber plantations. Only 0.6% of the country’s forested area is officially registered as customary territories for IP’ land tenure, but this rate should expand, with a potential to reach above 8% of the overall forested area in Indonesia. Private forests account for 7% of forested lands. Private forests are primarily looked after by smallholders and exploited by small-scale operators such as local farmers or farming cooperatives.

Regardless of property regime, SVLK certification is mandatory for all producers, traders and exporters of forest commodities in Indonesia. The system has evolved with time, but actors in different forest property regimes have experienced different incentives, levels of support, or challenges to adapt to such changes. We investigated such issues, which were reported in longer reads. ^109^ In this section, we highlight the differences for SVLK implementation in different forest regimes, and for different actors at the landscape level, before featuring a few policy recommendations.

### State concessions for industrial logging and plantations

Companies exploiting forest concessions perceive government support and the SVLK’s economic benefits to be moderate to weak. ^110^ Yet, concession holders have generally accepted and expressed satisfaction with SVLK rules, procedures and certification costs. For instance, holders of logging concessions were found to prefer the mandatory SVLK to voluntary mechanisms such as the FSC or the IFCC (Indonesian Forestry Certification Cooperation, which is part of PEFC, the Programme for Endorsement of Forest Certification). However, some companies still apply the voluntary mechanism to expand their international market. The SVLK is a government requirement for exporting, but companies obtain FSC certification as well to attract importers and buyers because the SVLK is less well known at international level. ^111^ In 2022, about half of state forest concessions were covered by SVLK certification. ^112^

### Customary forest for Indigenous Peoples

Customary forests are forest lands located in the territory of ‘customary law communities’, outside state forests. ^113^ By March 2022, Indonesia’s Ancestral Domain Registration Agency counted 17.6 million hectares (ha) of customary and indigenous territories that could potentially be recognized as such. ^114^ Yet, official recognition has been issued for about 16% of this potential (2.78 million ha), or 0.6% of Indonesia’s forested lands. Most customary forests were recognized by subnational authorities, ^115^ while the national government, through its Ministry of Environment and Forestry, had officially recognized 0.09 million ha as customary territories by April 2022. ^116^

With such low levels of recognition of land tenure for IP, their communities have enjoyed limited to no benefits from the SVLK. IP and local communities still struggle for autonomy, including the fight for land tenure recognition. ^117^ Moreover, even if they had land security, the SVLK would not accommodate the heterogeneity of IP and local communities. There are 1,331 ethnic groups recognized by the Indonesian government, ^118^ and not all IP want to be integrated into global supply chains. ^119^

### Private smallholding forests

Illegal logging in private smallholding forests is virtually unheard of, particularly when compared to illegal practices in state forest concessions. ^120^ Forest products and timber from private smallholding forests can be considered the easiest to control at farm level and the ‘most legal’ timber – when the land is inhabited and closely guarded and protected by smallholders or tenants.

Private forests represent 7% of Indonesia’s forested lands. The limited impacts of the SVLK on smallholder private forests had long been assumed, ^121^ and this was confirmed in our original field research between 2021 and 2022. Not surprisingly, the benefits of SVLK are not clearly perceived by smallholders. For instance, most smallholder farmers surveyed in Solo ^122^ either do not know what SVLK is (5% of 21 respondents) or have not perceived its impacts (76%). The respondents who reported SVLK benefits referred to a sense of pride (9%), commercialization facilities (5%) or better pricing for timber (5%). ^123^ For most smallholders, certification costs and complicated bureaucracies are burdensome, and they need to use the land in a way that is economical. Smallholders face challenges related to tree maintenance, capital and selling, so need more facilitation to cope with these matters than legal certification constraints.

Although smallholders are allowed to issue a SVLK self-declaration of conformity, ^124^ intermediaries have traditionally brokered declarations, according to focus group discussions and interviews. ^125^ This reduces the potential economic benefit that smallholders could draw from the SVLK.

### Final remarks

Indonesia’s FLEGT licensing scheme and SVLK are relatively effective in state forests, but considerably less so in private forests and virtually non-existent in customary forests. This profile of impacts has important distributive implications. Indigenous Peoples, local communities, artisanal producers and smallholders have not enjoyed benefits from the SVLK as much as medium to large operators of logging concessions and timber plantations.

Looking at the profile of benefits distributed, and the effectiveness of the SVLK, it is important to remember its origins and evolution, and to think about its future developments. First, with regards to the origins of the SVLK, it is important to highlight that foreign and international market mechanisms promoted by international agencies and donors have largely informed and motivated Indonesia’s forest policy reforms and integration into global supply chains. Second, on effectiveness, the SVLK has been associated with improving transparency and participation, but there are important misalignments between competing trade, forests policies and sustainable livelihoods. The SVLK took decades to develop (since at least the 2001 Bali Declaration on Forest Law Enforcement and Governance), but its impacts are still not significant enough to lead to more sustainable livelihoods. This has been a particular problem for the most marginalized citizens or populations of Indonesia, such as IP, local communities and smallholders. Third, we understand that the development of such regulations primarily serves the interests of traders, importers and actors in global supply chains, while not necessarily benefiting smallholder farmers (i.e. small private forests). All of this makes the glocalization of forest governance analysis and solutions an important avenue to be pursued.

Future policies and international partnerships should aim to promote sustainable land use and livelihoods, and seriously look at, and prioritize IP, local communities, and smallholders.

We recommend the maintenance of the full SVLK regime for state forests and large-scale concessions, and the creation of strategies and facilities crafted to benefit peoples and communities in private smallholding and customary forests. In the short term, facilities should be created to enable such groups to autonomously issue Self-Declaration of Conformity (SDoC) as legality and credibility assurance. But reforms in governance should go beyond the commodity market regime and observe the actual needs of forest peoples – to whom access to international markets is secondary to several other priorities. For instance, according to the interviews conducted in this research, the acceleration of recognition of customary territories is a priority for IP, as is better technical assistance, insurance and loans, eradication and prevention of pests and diseases, and facilitation of direct sales to the industry for smallholder farmers. Tackling these fundamental needs will help glocalize forest governance solutions and contribute to reducing poverty and inequality whilst safeguarding the climate and nature.

## Conclusions

By discussing the challenges in establishing equitable research partnerships between policy institutes across the Global North and Global South, and by featuring policy analysis and recommendations crafted between those institutes, this paper contributes to ideas and methods for the localization and glocalization of solutions and critiques of transnational mechanisms that affect land use, forest lands and peoples in the tropics. Glocalizing forest governance, in this project, implied advancing equitable research partnerships between think-tanks in the Global South and Global North, and strengthening a community of (policy research) practice for critical enquiry and engagement in partnerships for sustainable development.

A starting point was to identify the gaps between ideas of sustainability in international cooperation and localized practices. These have been problematic particularly when ‘solutions’ are crafted and decided away from producing sites and constituencies in the ‘developing’ world. Focal countries and firms in supply chains, primarily in the Global North, have struggled to understand the nuanced, cultural and context-specific issues and priorities in the Global South, and for the populations living in and around forested areas. The expansion of international and market mechanisms has reinforced unsustainable consumption and production patterns (which includes overconsumption in affluent societies) that are known to be underlying causes of deforestation, forest degradation, climate crises and inequality. In turn, institutions in the Global South have struggled for autonomy, for self-determination, for access to just and sustainable markets, and for resources to influence policymaking or conduct independent analysis. Acknowledging and confronting power dynamics of privilege and marginalization, which can be linked to historic colonization of lands and minds, is necessary in any serious enterprise such as international collaboration for sustainable development.

In this project, we have made efforts to tackle power asymmetries in the research partnership between think-tanks across continents, and to enable critical, glocal analysis of forest governance mechanisms, particularly when it comes to the investigation of their transnational origins, market underpinnings, achievements and challenges, participation profile, distributive impacts, lessons learned and recommendations to improve international partnerships.

As seen, the principal beneficiaries and the conditions for participation and inclusion vary by initiative, and by country. Notably, in the case of the Amazon Fund, the analysis led by Louise Nakagawa, Ariane Favareto, Tamara Tobias and Arilson Favareto showed that this REDD+ initiative contributed to certain non-monetary well-being indicators, as well as to participatory and more inclusive forums in Brazil. This differs from certain REDD+ experiences in other parts of the world, ^126^ and from the FSC certification for smallholders in the Brazilian Amazon. In the case of land-use regulations in the DRC, predictions that a moratorium on new logging concessions would not benefit the Congolese population, due to high levels of corruption, ^127^ was substantiated by the evidence communicated by Rigobert Minani, who also argues that foreign interventions focused on environmental protection have yet to acknowledge the social and development challenges in the region. In the case of Indonesia’s SVLK, the findings by Bramasto Nugroho, Damayanti Buchori, Fitta Setiajiati and Silfi Iriyani confirm what previous research had identified as a trend, i.e. legality verification systems favouring large companies ^128^ and export markets rather than smallholders and their needs and priorities. Overall, the case studies recognize limitations and challenges in influential North-South forest governance mechanisms – particularly when it comes to the distribution of costs and benefits – which is in line with the recent review by Delabre *et al*. ^129^

Additionally, the authors pointed to a set of alternatives for future forest solutions at the international level and at national level, thanks to the strengthening of their influence throughout the course of this project. For instance, all policy institutes had the chance to convene diverse stakeholders in forest governance, and to engage directly with ministers, representatives of marginalized communities, NGOs, entrepreneurs, as well as policy institutes in different continents. One of the lessons learned in the project is that well-crafted partnerships between policy institutes in the Global North and South contribute to enriching, strengthening and embedding transnational and glocal policy discussions at national and international forums. Investment in the establishment of equitable partnerships across regions and continents, and in learning by doing pedagogies (and other participatory action research methods) ^130^ can benefit future international cooperation programmes, particularly in the medium and long terms.

Alongside these procedural yet strategic remarks, donor entities and multilateral organizations would benefit from considering the following recommendations:

• Go beyond supporting legal or sustainable supply chains and their variants, such as in ‘green’, ‘carbon-neutral’, ‘net-zero’ or ‘deforestation-free’ supply chains. The focus on commodity markets has not shown clear benefits for those who are most dependent on tropical forests, and is unlikely to reduce excessive or unsustainable consumption levels in affluent societies, which is an underlying driver of deforestation, forest degradation, and the crises related to climate and inequality. Commodity-centred approaches need to be complemented by stronger environmental policies and regulations, coupled with mechanisms to guarantee human rights and the well-being of all. And this requires renewed theories of change, with greater scrutiny of assumptions, greater ambition for outcomes and impacts, and a strong commitment to learning strategies, as well as to Equality, Diversity and Inclusion mechanisms, through and beyond gender equality, that are wired towards just and sustainable livelihoods and outcomes.
• Work together with policy institutes across the Global South and Global North and develop context-specific, place-based mechanisms with strong participation of IP, local communities, with local knowledge, and with full consideration of local, subnational, national and regional development priorities. Market-based instruments created in the Global North and adapted or translated to ‘producing’ countries have not been a good fit to reverse unsustainable patterns of consumption and production. Co-creation and co-leadership are promising approaches for just and sustainable transitions, and must include equitable partnerships between institutions across the Global South and North. This is key to pave the way for the right kind (non-colonial forms) of glocalization that is needed in forest and land-use governance.
• Design and implement policy mixes and models to achieve global and domestic priorities for sustainable development. This entails balancing out different land uses (i.e. forestry, agriculture, mining, infrastructure) and sectoral policies between and within countries; regulation in domestic and international markets through fiscal, trade, environmental and agricultural policies; and a prioritization of not only poverty eradication, but the provision of multi-dimensional well-being and sustainable livelihoods as outcomes and impacts of international partnerships. ^131^ This relates to the importance of refreshing theories of change, and of promoting and pursuing diversification in land use and livelihood portfolios, which enable families and communities to be more resilient to shocks and disruptions.

These recommendations present the unequivocal need to build suitable and equitable forest governance mechanisms, through a glocalized approach, for future generations and the environment. The disconnect between theory and practice must be bridged, while mindful that resource governance involves multiple actors and interests at different scales, as well as all their social, ecological and location-specific complexities.

As it is the most central of all the Sustainable Development Goals, SDG 17 ‘global partnerships for sustainable goals’ must be improved and adapted to redress power imbalances between ‘global’ and ‘local’ imperatives, and between institutions across continents, in the Global North and in the Global South. Reflecting on the glocalization of development goals, and on the glocalization of partnerships for sustainable development will help with the design of innovative policy mixes and effective land-use and forest governance mechanisms.

### Final remarks

This report was built upon two needs that became opportunities. First, the need to rethink North–South research partnerships in international partnerships which required researchers to identify their own degrees of privilege in an unjust world and to redress power dynamics. Second, the need for more inclusive, participatory and transdisciplinary policy research, which would open up ways for diversity and plurality in the analysis of international policy mechanisms. These needs were taken as a springboard for innovation. We are proud of the collaborative approach adopted to produce this paper, and we invite serious consideration of the analysis and recommendations put forward by the authors in the Global South and the Global North to improve international partnership on land use, forests and sustainable livelihoods. The scale of this partnership is relatively small, but this could be scaled up to encompass other geographies and case studies, to promote diversity and mutual learnings in South–South and South–North–South (triangular) collaborations, and to keep advancing on equitable partnerships for sustainable development.

Transnational forest governance mechanisms need renovation. Market-based solutions and hybrid initiatives for ‘sustainable supply chains’ have been prominent since the 1990s, supported by public, private and hybrid initiatives and international partnerships focused on commodity exchange between Global South and Global North countries. These kinds of interventions can be improved and complemented by stronger environmental laws and public regulations (alongside their implementation) to raise standards that would effectively protect peoples and the environment, and contribute to the well-being of all. Notably, as discussed in this paper, innovation in policy instruments for land-use governance must position well-being and sustainable livelihood outcomes at the core of international partnerships, which in turn should be based on, and enhance, inclusive stakeholder participation.

## Data Availability

No data are associated with this article.

## Footnotes

^1^
Ometto, J.P. and Kalaba, F.K., (2022). CCP 7 Tropical Forests, Climate Change 2022: Impacts, Adaptation and Vulnerability *. Contribution of Working Group II to the Sixth Assessment Report of the Intergovernmental Panel on Climate Change*, https://doi.org/10.1017/9781009325844.024.2369.

^2^
Laumann, F., von Kügelgen, J., Kanashiro Uehara, T. H., & Barahona, M. (2022). Complex interlinkages, key objectives, and nexuses among the Sustainable Development Goals and climate change: a network analysis. *The Lancet Planetary Health*, *6*(5), e422–e430. https://doi.org/10.1016/s2542-5196(22)00070-5.

^3^
This collaboration was enabled by the Forest Governance, Legality and Climate (FGMC) programme of the UK Foreign, Commonwealth and Development Office (FCDO).

^4^
We refer to the definitions of these terms given by the Cambridge Dictionary, according to which partnership is ‘an agreement between organisations, people etc. to work together’, which differs from collaboration: ‘the situation of two or more people working together to create or achieve the same thing’; and cooperation: ‘the act of working together with someone or doing what they ask you’.

^5^
Led by TKU, FC and LN with inputs from the co-authors.

^6^
Led by CC with FC and TKU with inputs from the co-authors.

^7^
Led by LN, AF, TT and AF with inputs from the co-authors.

^8^
Led by RM with inputs from the co-authors.

^9^
Led by BN, FS, SI and DB with inputs from the co-authors.

^10^
Policy briefs available at http://forestgovernance.chathamhouse.org/publications/from-theory-to-practice-how-to-disrupt-north-south-research-partnerships

^11^
Hoare, A., & Kanashiro Uehara, T. (2022). Establishing fair and sustainable forest economies: Lessons learned from tackling illegal logging. *Research Paper, London: Royal Institute of International Affairs.* https://doi.org/10.55317/9781784135386.

^12^

Delabre, I., Boyd, E., Brockhaus, M., Carton, W., Krause, T., Newell, P., Wong, G. Y., & Zelli, F. (2020). Unearthing the myths of global sustainable forest governance. *Global Sustainability*, 3. https://doi.org/10.1017/sus.2020.11; Lambin, E. F., Meyfroidt, P., Rueda, X., Blackman, A., Börner, J., Cerutti, P. O., Dietsch, T., Jungmann, L., Lamarque, P., Lister, J., Walker, N. F., & Wunder, S. (2014). Effectiveness and synergies of policy instruments for land use governance in tropical regions. *Global Environmental Change*, 28, 129–140. https://doi.org/10.1016/j.gloenvcha.2014.06.007.

^13^
Koberg, E., & Longoni, A. (2019). A systematic review of sustainable supply chain management in global supply chains. *Journal of Cleaner Production*, *207*, 1084–1098. https://doi.org/10.1016/j.jclepro.2018.10.033.

^14^
REDD+ is a framework created by the UNFCCC COP to guide activities in the forest sector that reduce emissions from deforestation and forest degradation, as well as the sustainable management of forests and the conservation and enhancement of forest carbon stocks in developing countries.

^15^
‘Sustainable supply chain management’ tackles the integration of ‘environmental, social and economic goals across a focal firm’s supply chain processes’, or across a focal country or state, from the perspective of governments. Grabs, J., and Carodenuto, S.L. (2021), Traders as sustainability governance actors in global food supply chains: A research agenda, Business Strategy and the Environment, 30(2), pp. 1314-1332, https://doi.org/10.1002/bse.2686.

^16^
Wardell, A., Piketty, M. G., Lescuyer, G., & Pacheco, P. (2021). Reviewing initiatives to promote sustainable supply chains: The case of forest-risk commodities. *FTA Working Paper.* https://doi.org/10.17528/cifor/007944.

^17^
Hoare, A., & Kanashiro Uehara, T. (2022). Establishing fair and sustainable forest economies: Lessons learned from tackling illegal logging. *Research Paper, London: Royal Institute of International Affairs.* https://doi.org/10.55317/9781784135386.

^18^
Laumann, F., von Kügelgen, J., Kanashiro Uehara, T. H., & Barahona, M. (2022b). Complex interlinkages, key objectives, and nexuses among the Sustainable Development Goals and climate change: a network analysis. *The Lancet Planetary Health*, *6*(5), e422–e430. https://doi.org/10.1016/s2542-5196(22)00070-5.

^19^
Delabre *et al*. (2020), ‘Unearthing the Myths of Global Sustainable Forest Governance’.

^20^
Ibid., p. 4.

^21^
Shapiro-Garza, E., McElwee, P., Van Hecken, G., & Corbera, E. (2019). Beyond Market Logics: Payments for Ecosystem Services as Alternative Development Practices in the Global South. *Development and Change*, *51*(1), 3–25. https://doi.org/10.1111/dech.12546; Gómez-Baggethun, E., & Muradian, R. (2015). In markets we trust? Setting the boundaries of Market-Based Instruments in ecosystem services governance. *Ecological Economics*, *117*, 217–224. https://doi.org/10.1016/j.ecolecon.2015.03.016.

^22^
Schaafsma, M., Dreoni, I., Ayompe, L. M., Egoh, B., Ekayana, D. P., Favareto, A., Mumbunan, S., Nakagawa, L., Ngouhouo-poufoun, J., Sassen, M., Uehara, T. K., & Matthews, Z. (2022). A framework to understand the social impacts of agricultural trade. *Sustainable Development.* https://doi.org/10.1002/sd.2379; WCMC (2021), ‘What do we need to make trade more socially sustainable within exporting countries?’, Trade, Development and the Environment Hub blog, 25 November 2021, https://tradehub.earth/2021/11/25/what-do-we-need-to-make-trade-more-socially-sustainable-within-exporting-countries/.

^23^
Koberg, E., & Longoni, A. (2019). A systematic review of sustainable supply chain management in global supply chains. *Journal of Cleaner Production*, *207*, 1084–1098. https://doi.org/10.1016/j.jclepro.2018.10.033; Kanashiro Uehara, T., and O’Reilly K. (2022). Trade policy and sustainability: The WTO, the international fair trade charter, and five principles for system change. Manuscript submitted for publication.

^24^
Grabs, J., & Carodenuto, S. L. (2021b). Traders as sustainability governance actors in global food supply chains: A research agenda. *Business Strategy and the Environment*, *30*(2), 1314–1332. https://doi.org/10.1002/bse.2686.

^25^
Hasyim, Z., Laraswati, D., Purwanto, R. H., Pratama, A. A., & Maryudi, A. (2020). Challenges facing independent monitoring networks in the Indonesian timber legality assurance system. *Forest Policy and Economics*, *111*, 102025. https://doi.org/10.1016/j.forpol.2019.102025; Hoare, A. and Kanashiro Uehara, T. (2022), Forest sector revenues in Ghana, Liberia and the Republic of the Congo: The impact of reforms on collection and disbursement, Research Paper, London: Royal Institute of International Affairs, https://doi.org/10.55317/9781784135188.

^26^
Marije Schaafsma and others, ‘A Framework to Understand the Social Impacts of Agricultural Trade’, *Sustainable Development*, February, 2022, 1–13 < https://doi.org/10.1002/sd.2379>.

^27^
Marcello de Maria, Chris West, and others, ‘How Do We Link Local and National Level Measures with International Policy and Private Initiatives on Sustainable Trade for Agricultural Commodities?’, *Trade and Nature Discussion Papers*, 1.2 (2022), https://tradehub.earth/wp-content/uploads/2022/10/.

^28^
See, for instance, Meyfroidt, P., Roy Chowdhury, R., de Bremond, A., Ellis, E., Erb, K. H., Filatova, T., Garrett, R., Grove, J., Heinimann, A., Kuemmerle, T., Kull, C., Lambin, E., Landon, Y., le Polain de Waroux, Y., Messerli, P., Müller, D., Nielsen, J., Peterson, G., Rodriguez García, V., … Verburg, P. (2018). Middle-range theories of land system change. *Global Environmental Change*, *53*, 52–67. https://doi.org/10.1016/j.gloenvcha.2018.08.006.

^29^
For a classification of social orders of markets related to land use, see: Niederle, P. A. (2017). A pluralist and pragmatist critique of food regime’s genealogy: varieties of social orders in Brazilian agriculture. *The Journal of Peasant Studies*, *45*(7), 1460–1483. https://doi.org/10.1080/03066150.2017.1313238.

^30^
Gardner, T., Benzie, M., Börner, J., Dawkins, E., Fick, S., Garrett, R., Godar, J., Grimard, A., Lake, S., Larsen, R., Mardas, N., McDermott, C., Meyfroidt, P., Osbeck, M., Persson, M., Sembres, T., Suavet, C., Strassburg, B., Trevisan, A., … Wolvekamp, P. (2019). Transparency and sustainability in global commodity supply chains. *World Development*, *121*, 163–177. https://doi.org/10.1016/j.worlddev.2018.05.025.

^31^
Fletcher, R., Dressler, W., Büscher, B., & Anderson, Z. R. (2016). Questioning REDD+ and the future of market-based conservation. *Conservation Biology*, *30*(3), 673–675. https://doi.org/10.1111/cobi.12680; Delabre, I., Boyd, E., Brockhaus, M., Carton, W., Krause, T., Newell, P., Wong, G. Y., & Zelli, F. (2020). Unearthing the myths of global sustainable forest governance. *Global Sustainability*, 3. https://doi.org/10.1017/sus.2020.11.

^32^
Pokorny, B., Johnson, J., Medina, G., & Hoch, L. (2012). Market-based conservation of the Amazonian forests: Revisiting win–win expectations. *Geoforum*, *43*(3), 387–401. https://doi.org/10.1016/j.geoforum.2010.08.002.

^33^
Delabre *et al*. (2020), ‘Unearthing the Myths of Global Sustainable Forest Governance’.

^34^
Hoare, A., & Kanashiro Uehara, T. (2022). Establishing fair and sustainable forest economies: Lessons learned from tackling illegal logging. *Research Paper, London: Royal Institute of International Affairs.* https://doi.org/10.55317/9781784135386; Leach, M., & Scoones, I. (2013). Carbon forestry in West Africa: The politics of models, measures and verification processes. *Global Environmental Change*, *23*(5), 957–967. https://doi.org/10.1016/j.gloenvcha.2013.07.008.

^35^
Chatham House Forest Governance & Legality, http://forestgovernance.chathamhouse.org, country profiles for Brazil, DRC and Indonesia. For global, Ometto, J.P. and Kalaba, F.K., (2022). CCP 7 Tropical Forests, Climate Change 2022: Impacts, Adaptation and Vulnerability *. Contribution of Working Group II to the Sixth Assessment Report of the Intergovernmental Panel on Climate Change*, https://doi.org/10.1017/9781009325844.024.2369.

^36^
Forest Governance and Legality (2022). Country profiles. https://forestgovernance.chathamhouse.org/countries.

^37^
FAO & UNEP. (2020). *The State of the World’s Forests 2020.* www.fao.org. https://www.fao.org/state-of-forests/en/.

^38^
Global Forest Watch (2022). *Global Deforestation Rates & Statistics by Country.* https://www.globalforestwatch.org/dashboards/global/?category=summary. Underlying drivers of deforestation such as international and market forces, insecure land tenure, and the impacts of financialization (e.g., correlation of commodity prices with stock market dynamics rather than pure demand) are not included in this table. For a review on direct and underlying drivers of deforestation, see Ometto *et al*. (2022).

^39^
OPHI & UNDP. (2022). Unpacking deprivation bundles to reduce multidimensional poverty. In *Global Multidimensional Poverty Index 2022.* https://hdr.undp.org/system/files/documents/hdp-document/2022mpireportenpdf.pdf.

^40^
WID - Wealth and Income Database. (2018). *World: Top 10% net personal wealth share.* WID - World Inequality Database. https://wid.world/world/; Bajard, F., Chancel, L., Moshrif, R., & Piketty, T. (2022). Global Wealth Inequality on WID.world: Estimates and Imputations. The Source for Global Inequality. https://wid.world/document/global-wealth-inequality-on-wid-world-estimates-and-imputations-world-inequality-lab-technical-note-2021-16/. Net personal wealth share held by the p90p100 group. Net personal wealth is the total value of non-financial and financial assets (housing, land, deposits, bonds, equities, etc.) held by households, minus their debts. The personal or household sector – in the national accounts sense – includes all households and private individuals (including those living in institutions), as well as unincorporated enterprises whose accounts are not separated from those of the households who own them. The population is comprised of individuals over the age of 20. The base unit is the individual (rather than the household) but resources are split equally within couples.

^41^
Chatham House resourcetrade.earth. (2022). Commodity: Forestry products. Year: 2020. https://resourcetrade.earth/?year=2020&category=3&units=value&autozoom=1.

^42^
Ometto, J.P. and Kalaba, F.K., (2022). CCP 7 Tropical Forests, Climate Change 2022: Impacts, Adaptation and Vulnerability *. Contribution of Working Group II to the Sixth Assessment Report of the Intergovernmental Panel on Climate Change*, https://doi.org/10.1017/9781009325844.024.2369.

^43^
Forest Declaration Assessment Partners. (2022). Forest Declaration Assessment: Are we on track for 2030? Climate Focus (coordinator and editor). https://forestdeclaration.org/wp-content/uploads/2022/10/2022ForestDeclarationAssessment.pdf; Ozinga, S. & Hopkins, D. (2020), Tackling deforestation: the need for regulation. Forest Governance and Legality Blog, 10 August 2020. https://forestgovernance.chathamhouse.org/publications/tackling-deforestation-the-need-for-regulation .

^44^
Lambin *et al*. (2014). Effectiveness and synergies of policy instruments for land use governance in tropical regions. p. 137.

^45^
Lambin *et al*. (2014). Effectiveness and synergies of policy instruments for land use governance in tropical regions.

^46^
Ometto, J.P. and Kalaba, F.K., (2022). CCP 7 Tropical Forests, Climate Change 2022: Impacts, Adaptation and Vulnerability *. Contribution of Working Group II to the Sixth Assessment Report of the Intergovernmental Panel on Climate Change*, https://doi.org/10.1017/9781009325844.024.2369.

^47^
Tuck, E. & Young, K.W. (2012). Decolonization is not a metaphor. Decolonization: Indigeneity, Education & Society. Vol.1 (1).

^48^
Maselli, D., Jon-Andri, L., & Schmid, J. (2006). *Improving impacts of research partnerships.* Swiss Commission for Research Partnerships with Developing Countries KFPE. https://portal-cdn.scnat.ch/asset/1b8a6bc7-2840-5dcf-acb3-4bb086fbe718/KFPE_ImpactStudy.pdf?b=5e0dc39f-2adc-5ad2-96f0-92d97ba881b8&v=61b509e4-3355-5390-a481-5b57f6c1f20e_0&s=jvH2a27MJb7DLMjoA716qWITcPvxKy6yw4UGOQEl3f0xZzd-ApWDT_7Ow5IOSP5w-zXzkffEBzJyZOHDrp-uDAZv5cL3VHNe0bgpwsaOiYPbGnSpbYKVeirSF6YqfSaIT-JqT7IQZbb9KXOiQDkMKBAORnGrPuZwjjCKNLKk1Ec.

^49^
See, for instance, Forest Governance and Legality (2021). Forest voices: the path forward for strengthening forest governance. Forest Governance and Legality Blog. 14 October 2021. https://forestgovernance.chathamhouse.org/publications/forest-voices-the-path-forward-for-strengthening-forest-governance; Uehara, T. (2022). Takeaways from the Global Forum on Forest Governance 2022. Forest Governance and Legality Blog. 26 July 2022. https://forestgovernance.chathamhouse.org/publications/takeaways-from-the-global-forum-on-forest-governance-2022.

^50^
Arnstein, S. R. (1969). A Ladder Of Citizen Participation. *Journal of the American Institute of Planners*, *35*(4), 216–224. https://doi.org/10.1080/01944366908977225.

^51^
Arnstein, S. R. (1969). A Ladder Of Citizen Participation. *Journal of the American Institute of Planners*, *35*(4), 216–224. https://doi.org/10.1080/01944366908977225.

^52^
‘Positionality refers to the stance or positioning of the researcher in relation to the social and political context of the study—the community, the organization or the participant group. The position adopted by a researcher affects every phase of the research process, from the way the question or problem is initially constructed, designed and conducted to how others are invited to participate, the ways in which knowledge is constructed and acted on and, finally, the ways in which outcomes are disseminated and published.’ Bourke, B. (2014). Positionality: Reflecting on the Research Process. *The Qualitative Report.* https://doi.org/10.46743/2160-3715/2014.1026.

^53^
Bradley, M. (2017). Whose agenda? Power, policies, and priorities in North–South research partnerships. Development in Practice. Vol. 18, No. 6. http://www.jstor.org/stable/27751975; Mougeot, L. J. A. (2017). *Putting Knowledge to Work: Collaborating, Influencing and Learning for International Development.* Practical Action Publishing, IDRC.

^54^
Samuel S. 2019. Witsaja iki, or the good life in Ecuadorian Amazonia: Knowledge co-production for climate resilience. In: Ahearn A, Oelz M, Dhir RK (eds.) Indigenous Peoples and Climate Change: Emerging Research on Traditional Knowledge and Livelihoods. International Labour Organization.; Smith, H.A. 2007. “Disrupting the global discourse of climate change: The case of indigenous voices”, in M.E. Pettenger (ed.): The social construction of climate change: Power, knowledge, norms, discourses (Aldershot, Ashgate Publishing).

^55^
Samuel S. (2019). Witsaja iki, or the good life in Ecuadorian Amazonia: Knowledge co-production for climate resilience. In: Ahearn A, Oelz M, Dhir RK (eds.) Indigenous Peoples and Climate Change: Emerging Research on Traditional Knowledge and Livelihoods. International Labour Organization. https://ccafs.cgiar.org/resources/publications/witsaja-iki-or-good-life-ecuadorian-amazonia-knowledge-co-production.; Smith, H. A. (2006). Disrupting the Global Discourse of Climate Change: The Case of Indigenous Voices. In The Social Construction of Climate Change. Routledge. https://www.taylorfrancis.com/chapters/edit/10.4324/9781315552842-20/disrupting-global-discourse-climate-change-case-indigenous-voices-heather-smith .

^56^

http://forestgovernance.chathamhouse.org/publications

^57^
See Bent Flyvbjerg, ‘Five Misunderstandings About Case-Study Research’, *Qualitative Inquiry*, 12.2 (2006), 219–45 < https://doi.org/10.1177/1077800405284363>. The paradigmatic case is one of four modes of ‘information-oriented selection’ used to maximize the utility of information from small samples, and to develop a metaphor for the domain of transnational policy mechanisms that the cases concern.

^58^
Forest Stewardship Council. (2022). *Our History.* FSC United States. https://us.fsc.org/en-us/who-we-are/our-history .

^59^
Altruwood. (n.d.). *Brief History of FSC Certification.* Altruwood Blog. http://www.altruwood.com/brief-history-of-fsc-certification

^60^
Wardell D. and others, *Reviewing Initiatives to Promote Sustainable Supply Chains: The Case of Forest-Risk Commodities*, *FTA Working Paper*, 2021 < https://doi.org/10.17528/cifor/007944>.

^61^
Ortiz, F. (2018). Ten years on, Amazon Fund receives applause, criticism, faces new tests. *Mongabay Environmental News.* 21 December 2018. https://news.mongabay.com/2018/12/ten-years-on-amazon-fund-receives-applause-criticism-faces-new-tests/

^62^
Angelsen, A., Brown, S., Loisel, C., Peskett, L., Streck, C., & Zarin, D. (2009). Reducing emissions from deforestation and forest degradation (REDD): an options assessment report. In *CIFOR.* https://www2.cifor.org/gcs/modules/knowledge-sharing/redd-reducing-emissions-deforestation-forest-degradation/#:~:text=What%20is%20the%20history%20of,reducing%20carbon%20emissions%20from%20deforestation.

^63^
Garcia-Ulloa, J., & Koh, L. P. (2016). Payment for ecosystem services: the role of REDD + in primate conservation. *An Introduction to Primate Conservation*, 257–268. https://doi.org/10.1093/acprof:oso/9780198703389.003.0016

^64^
FAO & EFI. (2018). *Making forest concessions in the tropics work to achieve the 2030 Agenda: Voluntary Guidelines* (FAO Forestry Paper No. 180). https://www.fao.org/3/i9487en/I9487EN.pdf.

^65^
FAO & EFI. (2018). *Making forest concessions in the tropics work to achieve the 2030 Agenda: Voluntary Guidelines* (FAO Forestry Paper No. 180). https://www.fao.org/3/i9487en/I9487EN.pdf.

^66^
Harris, T. (2021). 5 Points to Understand Congo’s Logging Moratorium. *Greenpeace Blog.* 10 March 2021. https://www.greenpeace.org/africa/en/blogs/13258/5-points-to-understand-congos-logging-moratorium/#:~:text=Since%202002%2C%20a%20moratorium%20on,rampant%20in%20the%20logging%20sector.

^67^
OECD. (2020). *Towards Sustainable Land Use: Aligning Biodiversity, Climate and Food Policies, Chapter 5: Policy instruments relevant to sustainable land use.* OECD Publishing, Paris. https://www.oecd-ilibrary.org/environment/towards-sustainable-land-use_3809b6a1-en

^68^
Fishman, A., & Obidzinski, K. (2015). Verified Legal? Ramifications of the EU Timber Regulation and Indonesia’s Voluntary Partnership Agreement for the Legality of Indonesian Timber. *International Forestry Review*, *17*(1), 10–19. https://doi.org/10.1505/146554815814725095.

^69^
FSC Connect. (2022). *Facts & Figures: At a glance.* https://connect.fsc.org/impact/facts-figures .

^70^
UNFCCC. (2022). *National strategy Fact Sheet.* REDD+ Web Platform. https://redd.unfccc.int/fact-sheets/national-strategy.html.

^71^
Jonathan A. Foley, Gregory P. Asner, Marcos Heil Costa, Michael T. Coe, Ruth DeFries, Holly K. Gibbs, Erica A. Howard, Sarah H. Olson, Jonathan A. Patz, Navin Ramankutty, & Peter K. Snyder. (2007). Amazonia revealed: forest degradation and loss of ecosystem goods and services in the Amazon Basin. *Frontiers in Ecology and the Environment*, *5*(1), 25–32. https://doi.org/10.1890/1540-9295(2007)5; Strand, J., Soares-Filho, B., Costa, M. H., Oliveira, U., Ribeiro, S. C., Pires, G. F., Oliveira, A., Rajão, R., May, P., van der Hoff, R., Siikamäki, J., da Motta, R. S., & Toman, M. (2018). Spatially explicit valuation of the Brazilian Amazon Forest’s Ecosystem Services. *Nature Sustainability*, *1*(11), 657–664. https://doi.org/10.1038/s41893-018-0175-0.

^72^
Trancoso, R., Carneiro Filho, A., Tomasella, J., Schietti, J., Forsberg, B. R., & Miller, R. P. (2009). Deforestation and conservation in major watersheds of the Brazilian Amazon. *Environmental Conservation*, *36*(4), 277–288. https://doi.org/10.1017/s0376892909990373; Ometto, J. P., Aguiar, A. P. D., & Martinelli, L. A. (2011). Amazon deforestation in Brazil: effects, drivers and challenges. *Carbon Management*, *2*(5), 575–585. https://doi.org/10.4155/cmt.11.48; Leite-Filho, A. T., Soares-Filho, B. S., Davis, J. L., Abrahão, G. M., & Börner, J. (2021). Deforestation reduces rainfall and agricultural revenues in the Brazilian Amazon. *Nature Communications*, *12*(1). https://doi.org/10.1038/s41467-021-22840-7.

^73^
Heckenberger, M. J., Christian Russell, J., Toney, J. R., & Schmidt, M. J. (2007). The legacy of cultural landscapes in the Brazilian Amazon: implications for biodiversity. *Philosophical Transactions of the Royal Society B: Biological Sciences*, *362*(1478), 197–208. https://doi.org/10.1098/rstb.2006.1979; Fearnside, P. M. (2021). The intrinsic value of Amazon biodiversity. *Biodiversity and Conservation*, *30*(4), 1199–1202. https://doi.org/10.1007/s10531-021-02133-7.

^74^
Carneiro Da Cunha, M. & De Almeida, M. W. B. (1999). Indigenous people, traditional people, and conservation in the Amazon. *Daedalus*, *129*(2), 315–338; Graubard, S. R. (2000). *Brazil: The Burden of the Past; The Promise of the Future.* American Academy of Arts & Sciences. https://www.amacad.org/daedalus/brazil-burden-past-promise-future; Carneiro Filho, A., & Souza, O. B. (2009). *Atlas de pressões e ameaças às terras indígenas na Amazônia brasileira.* São Paulo: Instituto Socioambiental. https://repositories.lib.utexas.edu/handle/2152/17335; Yang, A. Toni, A. Waack, R. & Levy, J. (2021). Rethinking the Brazilian Amazon Sustainable development for a thriving future. Research Paper. Sustainability Accelerator. Chatham House Research Paper. https://www.chathamhouse.org/sites/default/files/2021-10/2021-10-21-rethinking-the-amazon-yang-et-al_0.pdf.

^75^
Campello, T. (Ed.). (2017). *Faces da Desigualdade no Brasil: Um olhar sobre os que ficam para trás.* CLACSO. https://doi.org/10.2307/j.ctvtxw2vg.

^76^
May P.H., Gebara M.F., Barcellos L. M., Rizek M., & Millikan B. (2016). The context of REDD+ in Brazil: Drivers, actors and institutions. 3rd Edition, CIFOR Occasional Paper 160. Bogor, Indonesia: CIFOR. https://doi.org/10.17528/cifor/006338.

^77^
Sabogal, C., Lentini, M., Pokorny, B., Silva, J.C., Zweede, J.C., Verissimo, A. & Boscolo, M. (2006). Manejo florestal empresarial na Amazônia brasileira: restrições e oportunidades: relatório síntese. *CIFOR.* https://www.cifor.org/publications/pdf_files/Books/BSabogal0601.pdf; Sparovek, G. (2009). E certificar, faz diferença? ? Estudo de avaliação de impacto da certificação FSC/RAS. https://www.imaflora.org/public/media/biblioteca/3_e_certificar_faz_diferenca.zip; Marcovitch, J., & Pinsky, V. C. (2014). Amazon Fund: financing deforestation avoidance. *Revista De Administração*, *49*(2), 280–290. https://doi.org/10.5700/rausp1146; Van Der Hoff, R., Rajão R., Leroy P., and Boezeman D. (2015). The parallel materialisation of REDD+ implementation discourses in Brazil. *Forest Policy and Economics.* Vol. 55(C). Pages 37-45. https://doi.org/10.1016/j.forpol.2015.03.005.

^78^
Fearnside, P.M. (2005). Deforestation in Brazilian Amazonia: History, Rates, and Consequences. *Conservation Biology.* Vol. 19 (3): 680–688. https://doi.org/10.1111/j.1523-1739.2005.00697.x; May P.H., Gebara M.F., Barcellos L. M., Rizek M., & Millikan B. (2016). The context of REDD+ in Brazil: Drivers, actors and institutions. 3rd Edition, CIFOR Occasional Paper 160. Bogor, Indonesia: CIFOR. https://doi.org/10.17528/cifor/006338; Silva, M. (2013). Políticas públicas e conservação dos recursos naturais: os aspectos socioambientais do programa bolsa floresta no modo de vida das comunidades ribeirinhas de Maués (AM). Dissertation (Master in Society and Culture in the Amazon). Universidade Federal do Amazonas, Manaus; Eula, M.J. (2017). O Fundo Amazônia e o desenvolvimento local sustentável: o caso da cooperativa de hortifrutigranjeiros de Boa Vista. Dissertation (Master in Regional Development), Universidade Federal de Roraima. Boa Vista; Marin, T.I.S, & Silva, L. (2019). Uma avaliação da eficácia operacional do Fundo Amazônia: um olhar crítico sob sua gestão. X Colóquio Organizações, Desenvolvimento e Sustentabilidade – CODS 2019, http://revistas.unama.br/index.php/coloquio/article/view/1851; Viergever, M., & Santos, P. (2019). Relatório de avaliação de Meio termo da Efetividade do Fundo Amazônia. http://www.fundoamazonia.gov.br/export/sites/default/pt/.galleries/documentos/monitoramento-avaliacao/5.avaliacoes-externas/FA-Relatorio-Distribuicao-de-Beneficios.pdf.

^79^
Cardoso, A.C., & Negrão, M.R.G. (2006). Considerações sobre a pobreza no Considerações sobre a pobreza no Brasil e suas manifestações nas Brasil e suas manifestações nas cidades da Amazônia. *Novos Cadernos NAEA.* vol.9, n. 1. pp. 95-118. ISSN 1516-6481; Campello, T. (Ed.). (2017). *Faces da Desigualdade no Brasil: Um olhar sobre os que ficam para trás.* CLACSO. https://doi.org/10.2307/j.ctvtxw2vg.

^80^
16 key-informant interviews were conducted. Details at https://www.cebrapsustentabilidade.org/assets/files/2022_PolicyBrief_CEBRAP_Nakagawa.pdf

^81^
For more information see: https://www.fundoamazonia.gov.br/en/home/

^82^
Which focuses on governance, institutions and the common use of natural resources. Theories of polycentric governance and inclusive coallitions, by Ostrom, Acemoglu, Robinson and Berdegué, for instance, were the theoretical backbone for the analysis.

^83^
Schaafsma, M., Dreoni, I., Ayompe, L. M., Egoh, B., Ekayana, D. P., Favareto, A., Mumbunan, S., Nakagawa, L., Ngouhouo-poufoun, J., Sassen, M., Uehara, T. K., & Matthews, Z. (2022b). A framework to understand the social impacts of agricultural trade. *Sustainable Development.* https://doi.org/10.1002/sd.2379.

^84^
Scoones, I. (2015). *Sustainable Livelihoods and Rural Development (Agrarian Change and Peasant Studies).* Fernwood Publishing. https://practicalactionpublishing.com/book/2123/sustainable-livelihoods-and-rural-development.

^85^
Minami, R. (2017). Unmet promises of extractive industries in Africa: corporate social responsibility: case study of Kitwe (Zambia) and Katanga (DR Congo). Nairobi, Kenya: Paulines Publications Africa. https://catalog.libraries.psu.edu/catalog/32173743.

^86^
Forest Governance and Legality (2022). DRC Country Profile. https://forestgovernance.chathamhouse.org/countries/democratic-republic-of-the-congo.

^87^
The Inspection Panel (2006), ‘Report and recommendations: Democratic Republic of Congo: Transitional Support for Economic Recovery Credit Operation (TSERO) (IDA Grant No. H 192- DRC) and Emergency Economic and Social Reunification Support Project (EESRSP) (IDA Credit No. 3824-DRC and IDA Grant No. H 064-DRC)’, Report No. 35192, The World Bank, https://documents1.worldbank.org/curated/en/112601468026108254/pdf/35192.pdf.

^88^
Long, C. (2011). Land Rights in the Democratic Republic of Congo: A New Model of Rights for Forest-Dependent Communities?. in *Land Struggles and Civil Society in Southern Africa.* pp. 1–28. https://mokoro.co.uk/wp-content/uploads/land_rights_in_the_DRC-2.pdf.

^89^
Trefon, T. (2007). Industrial logging in the Congo: is a stakeholder approach possible?. *South African Journal of International Affairs.* 13(2). pp. 101-114. https://doi.org/10.1080/10220460609556805.

^90^
Mulvagh, L. (2006). The Impact of Commercial Logging and Forest Policy on Indigenous Peoples in the Democratic Republic of Congo. *Indigenous Affairs.* 4. http://www.iwgia.org/graphics/Synkron-Library/Documents/publications/Downloadpublications/IndigenousAffairs/IA4-2006 articles/DRC.pdf.

^91^
Tshibwabwa Kuditshini, J. (2008). Global Governance and Local Government in the Congo: The Role of the IMF, World Bank, the Multinationals and the Political Elites’. *International Review of Administrative Sciences.* 74(2). pp.195–216. https://doi.org/10.1177/0020852308090773.

^92^
Masudi, E.B. (2022). DRC Pre COP27. https://drcprecop27.medd.gouv.cd/en/

^93^
Nakagawa, L., Favareto, A., Tobias, T. & Favareto, A. (2023). Forest governance in the Amazon and transition towards sustainable and inclusive use of natural resources: Lessons and challenges since FCS certification and the Amazon Fund. [Unpublished manuscript].

^94^
Ministère de l’Envirronnement et Développement Durable (2021). Le moratoire pour quel bénéfice 18 ans après. News. 29 September 2021, https://medd.gouv.cd/?s=moratoire.

^95^
Ir. José ILANGA LOFONGA, General director of forest in DRC ministry of environment Presentation during the national workshop July, 30, 2021.

^96^
Geopolis Hebdo (2021). COP 26 à Glasgow (Écosse): Félix Tshisekedi confirme le statut de pays-solution de la RDC’. 02 November 2021. https://www.geopolismagazine.net/cop-26-a-glasgow-ecosse-felix-tshisekedi-confirme-le-statut-de-pays-solution-de-la-rdc/.

^97^
Mavambu, R. (2021). Greenpeace Africa hands over a petition against the lifting of the moratorium with more than 100,000 signatories to the Prime Minister of the Democratic Republic of Congo. Greenpeace press release. 04 November 2021. https://www.greenpeace.org/africa/en/press/49456/greenpeace-africa-hands-over-a-petition-against-the-lifting-of-the-moratorium-with-more-than-100000-signatories-to-the-prime-minister-of-the-democratic-republic-of-congo/

^98^
Hoare, A. and Kanashiro Uehara, T.H. (2022). Establishing Fair and Sustainable Forest Economies: Lessons Learned from Tackling Illegal Logging. Research Paper. London: Royal Institute of International Affairs. https://doi.org/10.55317/9781784135386.

^99^
Democratic Republic of the Congo, Presidency of the Republic, General Inspectorate of Finance (2020), Rapport de mission relatif au contrôle de la légalité des allocations et cessions des concessions forestières et des droits dus au Trésor public par les exploitants forestiers formels [Mission report on the control of the legality of the allocations and transfers of forest concessions and rights due to the public treasury by formal loggers], https://medd.gouv.cd/wp- content/uploads/2022/04/Rapport-de-mission-relatif-au-controle-de-la-legalite-des-allocations-et-cessions-des-concessions-forestieres-et-des-droits-dus-au-Tresor-public-par-les-exploitants-forestiers-formels.pdf

^100^
Ibid.

^101^
Ibid.

^102^
Ibid.

^103^
Forest Governance and Legality (2022). DRC Country Profile. https://forestgovernance.chathamhouse.org/countries/democratic-republic-of-the-congo.

^104^
TEREA & S-FOR-S. (2016). *Evaluation of the EU FLEGT Action Plan (Forest Law Enforcement Governance and Trade) 2004-2014: Final Report Volume 1 (Main Report).* https://ec.europa.eu/environment/forests/pdf/FLEGT%20Eval%20Consultant%20Report%20EN.pdf; Hoare, A., & Kanashiro Uehara, T. (2022). Establishing fair and sustainable forest economies: Lessons learned from tackling illegal logging. *Research Paper, London: Royal Institute of International Affairs.* https://doi.org/10.55317/9781784135386.

^105^
Hoare, A., & Kanashiro Uehara, T. (2022). Establishing fair and sustainable forest economies: Lessons learned from tackling illegal logging. *Research Paper, London: Royal Institute of International Affairs.* https://doi.org/10.55317/9781784135386.

^106^
Fourteen other countries were negotiating or preparing to implement similar schemes in 2022, but Indonesia remained the only one with an operational licensing scheme. FLEGT Independent Market Monitor, Monitoring board, https://stats.flegtimm.eu (last access 19 September 2022)

^107^
SVLK abbreviation was revised, whereby ‘K’ no longer meant *Kayu* (timber), but rather *Kelestarian* (sustainability).

^108^
Nugroho B., and Tiryana T. 2013. Implications of the private property right to the community forest business formalization through the certification policy. Jurnal Manajemen Hutan Tropika XIX (3): 178 – 186. Maryudi A, Laraswati D, Sahide MAK, Giessen L. 2021. Mandatory legality licensing for exports of Indonesian timber products: balancing the goals of forest governance and timber industries. Forest Policy and Economics 124: 102384. Doi: https://doi.org/10.1016/j.forpol.2020.102384. Villanueva, F.D.P., Tegegne Y.T., Winkel, G, Cerutti P.O., Ramcilovic-Suominen S., McDermott C.L, Zeitlin J., Sotirov, M., Cashore, B., Wardell, D.A., Haywood A., Giessen, L. 2022. Effects of EU illegal logging policy on timber-supplying countries: A systematic review. Journal of Environmental Management 327 (2023) 116874

^109^
Data was collected from July 2021 to June 2022, through a combination of interviews, focus group discussions, field visits, observations, and three workshops. Approximately 317 participants were involved as respondents and informants, representing state forest licence holders, smallholder private forests, and customary forests. Nugroho, B., Buchori, D., Setiajiati, F. & Iriyani, S. (2022). Effectiveness of SVLK Implementation in Forest Property Rights Regimes Following Indonesia's Omnibus Law: An Institutional Economic Perspective. [Unpublished manuscript].

^110^
Survey results of 11 natural forest concessions and nine monoculture timber plantation concessions. Results were confirmed by participants in the second national workshop on 19 April 2022.

^111^
Nurkomariyah S, Firdaus M, Nurrochmat DR, Erbaugh JT. (2019). Questioning the competitiveness of Indonesian wooden furniture in the global market. *IOP Conf. Series: Earth and Environmental Science.* 295. https://doi.org/10.1088/1755-1315/285/1/012015.

^112^
Indonesia has 262 units of natural forest concessions and 293 units of monoculture timber plantation concessions (MoEF website phl.menlhk.go.id/infografis, accessed on 20 September 2022). The total concessions that received the SVLK up to September 2022 were 178 units for natural forest and 152 units for monoculture timber plantation (informed by MoEF Staff at the Directorate General of Sustainable Forest Management). Indonesia's production forests cover 68.82 million ha. Notably, the area of natural forest concessions declined by 69% while industrial plantations increased by 142% in the past two decades. This change can be explained by the political reforms in Indonesia in 1998 and international political economy pressures, which have enabled the expansion of monocultural timber plantations.

^113^
The customary law community is a traditional community in association with institutions and instruments of customary law, which is still adhered to and which still relates to collecting forest products in the surrounding forest areas, whose existence is confirmed by regional regulations (Regulation of MoEF No. 9 the Year 2021). Previously, the customary forest was part of the state forest (Law no. 41 the Year 1999), no longer has that status since the Constitutional Court decision no. 35/PUU-X/2012.

^114^
Working Group for ICCAs in Indonesia. (2022, April 22). A five-million-hectare increase in the registered Indigenous territory in Indonesia: New BWRA data. *ICCA Consortium.* https://www.iccaconsortium.org/index.php/2022/04/12/indonesia-indigenous-peoples-territory-record-update-bwra/.

^115^
Working Group for ICCAs in Indonesia. (2022, April 22). A five-million-hectare increase in the registered Indigenous territory in Indonesia: New BWRA data. *ICCA Consortium.* https://www.iccaconsortium.org/index.php/2022/04/12/indonesia-indigenous-peoples-territory-record-update-bwra/.

^116^
Jong, H. N. (2022). Indonesian government lagging independent effort to recognize Indigenous lands. *Mongabay Environmental News.* 05May 2022. https://news.mongabay.com/2022/05/indonesian-government-lagging-independent-effort-to-recognize-indigenous-lands/.

^117^
Jong, H. N. (2022). Indonesian government lagging independent effort to recognize Indigenous lands. *Mongabay Environmental News.* 05May 2022. https://news.mongabay.com/2022/05/indonesian-government-lagging-independent-effort-to-recognize-indigenous-lands/.

^118^
Badan Pusat Statistik. “Informasi Terbaru.” 2021. https://bps.go.id/

^119^
Uehara, T. (2022). Takeaways from the Global Forum on Forest Governance 2022. Forest Governance and Legality Blog. 26 July 2022. https://forestgovernance.chathamhouse.org/publications/takeaways-from-the-global-forum-on-forest-governance-2022.

^120^
Nugroho B, Buchori D, Iriyani S, Setiajiati F. (2022). Efektivitas penerapan SVLK pada Berbagai Tipe Alas Hak. Policy Brief Pertanian, Kelautan, dan Biosains Tropika. Vol 4 No. 3. https://dpis.ipb.ac.id/wp-content/uploads/2022/08/12.-Efektivitas-Penerapan-SVLK-pada-Berbagai-Tipe-Alas-Hak.pdf.

^121^
Nurrochmat, D.R., Dharmawan, A.H., Obidzinski, K., Dermawan, A., and Erbaugh, J.T. (2016), Contesting national and international forest regimes: Case of timber legality certification for private forests in Central Java, Indonesia, *Forest Policy and Economics,* 68: 54 – 64, https://doi.org/10.1016/j.forpol.2014.09.008.

^122^
The survey and focus group discussion are part of this study, conducted in Solo Raya on 17 March 2022

^123^
Findings from FGD Solo on 17 March 2022

^124^
Ministerial Regulation Permenhut 8, 2021.

^125^
Nugroho B, Buchori D, Iriyani S, Setiajiati F. (2022). Efektivitas penerapan SVLK pada Berbagai Tipe Alas Hak. Policy Brief Pertanian, Kelautan, dan Biosains Tropika. Vol 4 No. 3. https://dpis.ipb.ac.id/wp-content/uploads/2022/08/12.-Efektivitas-Penerapan-SVLK-pada-Berbagai-Tipe-Alas-Hak.pdf.

^126^
Ece M. (2021). Creating property out of insecurity: territorialization and legitimation of REDD+ in Lindi, Tanzania. *The Journal of Legal Pluralism and Unofficial Law.* Volume 53. https://doi.org/10.1080/07329113.2021.1900512.

^127^
Cannon, J. (2018). NGOs seek suspension of forest-related funding to DRC in response to proposed end to logging moratorium. Mongabay Series: Global Forests. 08 March 2018. https://news.mongabay.com/2018/03/ngos-seek-suspension-of-forest-related-funding-to-drc-in-response-to-proposed-end-to-logging-moratorium/.

^128^
Maryudi, A., Acheampong, E., Rutt, R.L., Myers, R. and McDermott, C.L. (2020). “A Level Playing Field”? – What an Environmental Justice Lens Can Tell us about Who Gets Leveled in the Forest Law Enforcement, Governance and Trade Action Plan. *Society & Natural Resources.* 33(7), pp. 859-875. https://doi.org/10.1080/08941920.2020.1725201.

^129^
Delabre, I., Boyd, E., Brockhaus, M., Carton, W., Krause, T., Newell, P., Wong, G. Y., & Zelli, F. (2020). Unearthing the myths of global sustainable forest governance. *Global Sustainability*, 3. https://doi.org/10.1017/sus.2020.11.

^130^
See, for instance, David Coghlan and Mary Brydon-Miller, ‘Paulo Freire’, in *The SAGE Encyclopedia of Action Research*, ed. by David Coghlan and Mary Brydon-Miller (London: SAGE Publications, 2014), pp. 368–71 <https://doi.org/10.4135/9781446294406>.

^131^
See, for instance, Schaafsma and others; Ian Scoones, *Sustainable Livelihoods and Rural Development* (Rugby, UK: Practical Action Publishing, 2015); Marcello de Maria, Ilda Dreoni, and others, *What Do We Need to Make Trade More Socially Sustainable within Exporting Countries?*, Discussion Paper, 2021 <https://tradehub.earth/wp-content/uploads/2021/11/TRADE_Discussion_Paper_5.pdf>. rooks, S., Nicholas, H., West, C., De Maria, M., and Komarudin, H. (2022), *Taking responsibility for supply chain impacts: who, why and how?,* Discussion Paper 6, Chatham House, UNEP and WCMS, https://tradehub.earth/wp-content/uploads/2022/03/FAQ6-3-003.pdf.
