# Supplementary material for: ‘Glocalizing’ land-use and forest governance in the tropics: examining research partnerships and international forest policies affecting Brazil, DRC and Indonesia
Source: F1000Res. 2023 Feb 2;12:125. [Version 1] doi: 10.12688/f1000research.130219.1 (PMC10338982; doi:10.12688/f1000research.130219.1)
Supplement: Peer review history

# Peer review: Reviewer response for version 1

**DOI:** 10.5256/f1000research.142956.r176883

**Published:** 2023-07-12

**Authors:** Krossy Mavakala

**Affiliations:** 1 Department of Political and Social Sciences, Universite Pedagogique Nationale, Kinshasa, Kinshasa, Democratic Republic of the Congo

**Summary**

 The article addresses a crucial and topical issue about forest governance in the tropics under the optique of examining partnerships and international forest policies that affect Brazil, the Democratic Republic of Congo and Indonesia. As an introduction, the authors stated that international and market forces are key underlying drivers of deforestation and degradation of forest. Then, the authors call the international community to take stock of how it frames and manages land-use, forest and climate policy collaborations. International collaboration needs to be led in an inclusive manner including revisiting SDGs (Sustainable Development Goals) on ‘Global Partnerships for Sustainable Development’. Authors define ‘global’ or ‘globalizing’ as a way to redress imbalances in the interplay between ‘global’ and ‘local’ imperatives in international collaboration, and between institutions in Global North and Global South countries. The article is a result of a twenty-month collaborative project between Chatham House from the UK, CEBRAP (Centro Brasileiro de Análise e Planejamento) from Brazil, CEPAS (Centre d’Etudes pour l’Action Sociale) from the DRC and CTSS (Centre for Transdisciplinary and Sustainability Sciences) from Indonesia. The project aimed to establish and discuss equitable research partnerships while examining prominent transnational initiatives affecting the forest lands and peoples in the tropics.

 The article is subdivided into three big parts each with sub-part. Apart from the introduction and the conclusion, the first part of the article talks about Forest governance solutions that need a critical analysis, the second one is about North–South and South–South research partnerships emphasing setting of relationships between research partners while underlying the methodology and the outputs of the research, and the last part is about (G)local views on transnational policy instruments with regard to revisiting community and governance impacts of the FSC and Amazon Fund in Brazil, Regulating logging concessions and the moratorium in the DRC, and Verifying legality in different forest regimes in Indonesia. The article has shown interesting results in the three countries and different domains of reflexions through all the countries object of the study.

***1. Revisiting community and governance impacts of the FSC and Amazon Fund in Brazil***

 The situation of uncontrolled and illegal use of natural resources in Brazil that made the biome suffer severely has been reversed since 2012 until the deforestation become greater recently with impacts on poverty of people from the Amazon region. Therefore, the section about revisiting community and governance impacts of the FSC and Amazon Fund in Brazil aims to revisit two mechanisms affecting land use and livelihoods such as the Forest Stewardship Council (FSC) and the Amazon Fund as Redd+ mechanism. The first mechanism is about promoting better forest management practices while the second one is about reducing emissions and carbon markets on payment for environmental services.

 It appeared in the context of Brazil that the FSC mechanism had several limitations particularly for smallholders, Indigenous Peoples and local communities as it depends on external support, generally absent. The survey conducted with minoritiy groups shows that smallholders face constant threats and incursions by land grabbers, that makes it challenging for minority groups to obtain and maintain FSC certification. In the other hand, the Amazon Fund contributed to strengthen environmental agencies at the state and federal levels. The legacy of the Amazon Fund was noticeable, particularly by including women and traditional communities, even when unfavorable political decisions were applied.

 Therefore, authors recommended not to consider FSC certification and the Amazon Fund as unique and sufficient solutions; but, to also discuss about new strategies and actions that governments, funders and investors could enact.

***2. Regulating logging concessions and the moratorium in the DRC***

 Authors talk about the moratorium on the allocation of new industrial logging concessions, a process that begun in 2002 and that the government wanted to lift in 2021. Shown as a way to put an end to what Congolese government calls a widespread of looting, exploitation, and destruction of the country, the moratorium itself turned out to be both an opportunity and a failure. Established by a presidential decree in 2015, the moratorium presented three conditions such as the conversion of old logging titles into forest concession contracts, deployment of transparency mechanisms on forest concessions and plan a phased lifting of the moratorium. The lifting of the moratorium has been announced in 2021 while the conditions were not fulfilled, government claiming the DRC as a « climate crisis solution country ». Therefore, authors of the article examined the views of different stakeholders about the moratorium on industrial logging concessions in order to find ways forward in terms of national and international policy work.

 One of the views from the government shows that maintaining a 20-year old moratorium is irresponsible emphasizing the potential for land resources to contribute to revenue collection for the government, to the development of infrastructure, and to the benefit of people. International NGOs and the Congolese civil society opposed to this point of view the contradiction between the DRC’s climate and forest commitments and its plan to lift the moratorium on new logging concessions, underlying the fact that the main beneficiaries of such a decision would certainly be foreign companies, and not necessarily Congolese communities. Therefore, Congolese government has been asked to firstly design and define a phased programme to progressively lift the moratorium. 

 DRC’s forest law enforcement has been said weak by the authors, DRC’s administration having granted contracts through bilateral negotiations between entrepreneurs and government officials that were unlawful, lacked transparency, and fairness. Views from the DRC government said that logging concessions were linked to corruption and benefited personal interests. The capacity of the country to enact reforms or enforce regulations shown numerous challenges for the public service.

***3. Verifying legality in different forest regimes in Indonesia***

 Several International Organization including the World Bank, the United Nations Forum on Forests, the Group of Eight, the EU, and the Association of South-East Asian Nations have supported multilateral frameworks to improve forest law enforcement, governance and associated trade with the aim to reduce illegal logging and the related trade that would halt deforestation and foster sustainable forest management with negative impacts on rural livelihoods and sustainable development.

 Being the first country in the world to have operationalized the Forest Law Enforcement, Governance and Trade (FLEGT) licensing scheme supported by the SVLK (Sistem Verifikasi Legalitas Kayu), the SVLK had several changes whose the major one is the 2021 *Omnibus Law* on job creation that broadened the scope of the system, to encompass verifications on sustainability, and to cover all timer and non-timber products, and all forest property regimes. The section talks about the effectiveness of the SVLK for Indonesia in different regimes such as the state, customary and private forests.

 While most forested areas belong to the State, with a lesser quantity to the indigenous peoples and a fewer one to privates, companies exploiting concessions perceive government support and the SVLK’s economic benefits to be moderate or weak. Holders, therefore, preferred the mandatory SVLK to voluntary mechanisms like the FSC or the IFCC.

 Authors give this final remark that licensing scheme and SVLK have relative effect in state forests while effects are minor in privates and non-existent in customary forests with negative implications in Indigenous Peoples, local communities, artisanal producers and smallholders.

 Now, it a place to assess the article by answering to some questions that the F1000Research have organized in a way of peer-reviewing efficiently this work. Here are questions:

- ***Is the work clearly and accurately presented and does it cite the current literature?***

Yes, the work is clear and accurately presented and cited current literature. However, we suggest that the authors give a sufficient place to words from the interviewees as the study has made more than 20 months and included several researchers from several research institutions. Some pages of the article have had a half of citations while we do not see anywhere some quotes showing that what authors say comes from people from the field. Without showing that it is a field study, we are afraid that readers think it is a work done from the library. Our advice is to ask authors to reduce the amount of citations and give more insights and importance to views from people from the field.

 In the presentation of the work, we observe that the authors have tried to advise others like the international community, that we think could lead to a certain risk of *ethnocentrism* or *egocentrism *and lead to a feeling of everything the international community has done is null seen from the Global South. We all know that a lot has been done while a lot has to be done in the future. Sentences like « the international community needs to take stock of how it frames and manages land-use, forest and climate policy collaborations… » or « a rethink of international collaborations needs to be conducted in an inclusive manner » looks like either the international community does not know what they are doing or efforts that the international community have made for decades were not well designed, and, therefore, once they act for advice from authors, everything will drive better. We know that it is useful, or it is a fashionable way of characterizing the international community, but, we think that we should be grateful to what we all have done in the side of conservation to make it move better, even though we know that challenges are still numerous to face. It should be important that we design accurate ways of assessing efforts made since then through metrics that could easily show improvements made in the sector of conservation. And, what does the international community mean for those three countries when the DRC is an international community member when we talk about Brazil and Indonesia, vice-versa. It is obvious that numerous works discuss the role of institutions such as the Word Bank, the IMF, the EU, the UN, and so on in designing programmes that could impact the global challenges. And, the inclusive aspect of the conservation authors seek to be included in the conservation is already elements of the discourse of the international community since decades, and participation of international, national and local communities in efforts to undertake global climate change is not aspect to be ignored. However, it is also obvious that numerous authors, institutions and countries have criticized the way those institutions function. It is however obvious that those institutions and their programmes have impacted the world, including the way we organize conservation responses. What was positive and what is negative are things to be assessed with objectivity. From then, there's still a long way to go.

 Our point of view remains when authors talk about Global South governments. Some sentences address the functioning of governments from the South when it is to say like « there is no political and policy coordination to reconcile risks and opportunities between urbanization, rural development, food, energy and climate security ». I think those are extreme affirmations that could be revisited or relativized as governments from the South have also made sufficient efforts to participate in the fight of sustainable environment.

- ***Is the study design appropriate and is the work technically sound?***

Yes, the study is appropriate and the work sounds technically. I was asking myself what was the transition between both parts of the work that are « Forest governance solutions » and « (G)local views on transnational policy instruments **»**. Authors succeeded in explaining what is the link between those parts: « four institutes sought to establish and discuss equitable research partnerships while examining prominent transnational initiatives that are affecting forest lands and peoples in the tropics ».  However, some questions raise when it is to question the status, functions and rules of members of those institutes according to their involvement in actions and programmes that affect forest lands and peoples of the three countries? Are they just researchers from independent research center or are they government members with related connections to decision-making about forest lands and peoples? In this part, we also question the role of Chatham House in designing the survey. It looks like authors are talking about equitability while Chatham House remains « above » other partners in the research. Indeed, Chatham House has looked for funds to finance the research and has designed a questionnaire that was implemented to researchers in the Global South. It is Chatham House that controlled the budget, that makes it not acting as a partner, but a coordinator of the project with a risk to negatively the equitability criterion it fights for. Authors do not state that either researchers from the South either contributed to budget or researchers from the North responded to the questionnaire. By not doing so, equitability between Global North and Global South remains in a risk of maintaining the imbalance between partners.

- ***Are sufficient details of methods and analysis provided to allow replication by others?***

Partly. The section on methodology leaves us wanting more. The methodology is not precise about what kind of method (and techniques) were used as in the beginning authors talk about a questionnaire submitted to researchers from the Global South and some focus groups and interviews so far. Author sare not precise if it was quantitative, qualitative or mixed approach of the survey that led to the results that we are acknowledging today. Authors do not talk a lot about the quality of the interviewees, their functions in their respective institutions, their sociological profile, and so on. We don’t know how many researchers they questioned and how many times they had in produce the data and we don’t know if the survey was led in the Global South or in the Global North or if it was an online one. I think some precisions are important to help the survey to benefit from a replication if possible. Being precise about how they produced the data could help why they have chosen to present their results in that way, as I have underlined that those results look like coming from a huge literature source than from a long time field work survey. We need some quotes and some sentences and sociological profiles from people from the ground to make the article feed the voices of people from the Global South.

- ***If applicable, is the statistical analysis and its interpretation appropriate?***

Not applicable. The style or the methodology applied by the authors have not asked them to use sufficient statistical analysis, which in no way detracts from the quality and nature of the information contained in this article.

- ***Are all the source data underlying the results available to ensure full reproducibility?***

Yes

- ***Are the conclusions drawn adequately supported by the results?***

Yes

Is the work clearly and accurately presented and does it cite the current literature?

Yes

If applicable, is the statistical analysis and its interpretation appropriate?

Not applicable

Are all the source data underlying the results available to ensure full reproducibility?

Yes

Is the study design appropriate and is the work technically sound?

Yes

Are the conclusions drawn adequately supported by the results?

Yes

Are sufficient details of methods and analysis provided to allow replication by others?

Partly

Reviewer Expertise:

Social and Politicl Science & Forests and Tropical Lands Management

I confirm that I have read this submission and believe that I have an appropriate level of expertise to confirm that it is of an acceptable scientific standard, however I have significant reservations, as outlined above.

# Peer review: Reviewer response for version 1

**DOI:** 10.5256/f1000research.142956.r176892

**Published:** 2023-07-04

**Authors:** Felipe Nunes

**Affiliations:** 1 Universidade Federal de Minas Gerais, Belo Horizonte, State of Minas Gerais, Brazil

A well-written manuscript that addresses an important perspective for better understanding the failure of transnational and market initiatives to effectively combat deforestation in the tropics. Small comments and suggestions to the authors above (and highlighted in the pdf document shared). 

 I suggest citing the European Union Regulation on deforestation-free imports recently approved and especially discussing how it is shaping EU-Mercosul Agreement, which once signed could become the world's largest free trade agreement: https://environment.ec.europa.eu/topics/forests/deforestation/regulation-deforestation-free-products_en; https://www.greens-efa.eu/en/article/study/the-eu-mercosur-free-trade-agreement-its-impacts-on-agriculture

 It is important to discuss the role of private certifications as it they have contributed to expanding market-based solutions despite several cooperative scandals and evidence of insufficient progress toward achieving anti-deforestation targets and greenhouse gas emissions reductions ^1^.

 Governments can also play an important role in providing transparency in high-risk supply chains by addressing compliance with socio-environmental legislation at the farm level. 

 I suggest the authors include 2 or 3 sentences indicating ways through boundary work and boundary objects literature ^2^- ^4^. 

 I suggest that the authors also cite critical studies on the effectiveness of the Amazon Fund, e.g., Correa et al., (2019) ^5^.

Is the work clearly and accurately presented and does it cite the current literature?

Partly

If applicable, is the statistical analysis and its interpretation appropriate?

Not applicable

Are all the source data underlying the results available to ensure full reproducibility?

No source data required

Is the study design appropriate and is the work technically sound?

Yes

Are the conclusions drawn adequately supported by the results?

Yes

Are sufficient details of methods and analysis provided to allow replication by others?

Yes

Reviewer Expertise:

Environmental science, Geoscience and Spatially-explicit modeling

I confirm that I have read this submission and believe that I have an appropriate level of expertise to confirm that it is of an acceptable scientific standard, however I have significant reservations, as outlined above.

## References

[1] : The rotten apples of Brazil's agribusiness. *Science* .2020;369(6501) : 10.1126/science.aba6646 246-248 10.1126/science.aba6646 32675358

[2] : Amplifying Indigenous community participation in place-based research through boundary work. *Progress in Human Geography* .2019;43(6) : 10.1177/0309132518807758 1020-1043 10.1177/0309132518807758

[3] : Boundary work in climate policy making in Brazil: Reflections from the frontlines of the science-policy interface. *Environmental Science & Policy* .2016;59: 10.1016/j.envsci.2016.02.009 85-92 10.1016/j.envsci.2016.02.009

[4] : Exploring the role of boundary work in a social-ecological synthesis initiative. *Journal of Environmental Studies and Sciences* .2023;13(2) : 10.1007/s13412-022-00811-8 330-343 10.1007/s13412-022-00811-8

[5] : Amazon Fund 10 Years Later: Lessons from the World’s Largest REDD+ Program. *Forests* .2019;10(3) : 10.3390/f10030272 10.3390/f10030272

# Peer review: Reviewer response for version 1

**DOI:** 10.5256/f1000research.142956.r176890

**Published:** 2023-06-30

**Authors:** Peter Kanowski

**Affiliations:** 1 Fenner School of Environment and Society, Australian National University, Canberra, Australian Capital Territory, Australia

I acknowledge the spirit in which the authors and institutions have undertaken and reported this work, and commend them for that. One could not argue with the intentions of strengthening the agency of institutions of the Global South in collaborative research, or of South-South collaboration; and, from the evidence in the paper, it appears that the project has been successful in doing so.  

 Such models of research partnership are not new, of course (albeit underrepresented); Australia's ACIAR (www.aciar.gov.au) was established 40 years ago to support research between one country of the Global N and a cohort of those of the Global S. Its original focus on primarily technical issues has broadened over the past decade to a stronger policy focus. Some studies have explored the factors that contribute to the success, or otherwise, of these partnership projects (e.g., Bartlett 2018 ^1^).

 The Brazilian and DRC case studies reported here each recommend inclusive, transparent multistakeholder forums as platforms for addressing the forest governance challenges they explore. Such platforms exist, of course, internationally (The Forests Dialogue; theforestsdialogue.org) and in particular countries (e.g., Brazil; dialogoflorestal.org.br). 

 Methodologically, more information on the actual form of the institutional analysis conducted in Brazil. The analytical approaches adopted in DRC and Indonesia were not clear to me.

 For the paper as a whole, the results reiterate those of the wider literature (eg Katila et al 2020, DOI: 10.1017/9781108765015 ^2^). While I don't disagree with the emphasis in the conclusions on reimagining and reforming N-S relationships, I am reminded by each of the case studies of how so much of forest governance outcomes is determined by domestic political economy, which is influenced only to some extent by the N-S dynamics. It would be helpful to see some further thoughts on strategies for change in that arena emerge from each of the cases.

Is the work clearly and accurately presented and does it cite the current literature?

Yes

If applicable, is the statistical analysis and its interpretation appropriate?

Not applicable

Are all the source data underlying the results available to ensure full reproducibility?

No source data required

Is the study design appropriate and is the work technically sound?

Yes

Are the conclusions drawn adequately supported by the results?

Partly

Are sufficient details of methods and analysis provided to allow replication by others?

Partly

Reviewer Expertise:

Forest governance

I confirm that I have read this submission and believe that I have an appropriate level of expertise to confirm that it is of an acceptable scientific standard.

## References

[1] : Understanding and evaluating success in international forestry research projects: experience from ACIAR projects in Vietnam, Indonesia and Papua New Guinea. *International Forestry Review* .2018;20(3) : 10.1505/146554818824063096 274-295 10.1505/146554818824063096

[2] Sustainable Development Goals: Their Impacts on Forests and People.2019; 10.1017/9781108765015 10.1017/9781108765015
